# Shortened lifespan induced by a high-glucose diet is associated with intestinal immune dysfunction in *Drosophila sechellia*

**DOI:** 10.1242/jeb.244423

**Published:** 2022-10-31

**Authors:** Maiko Abe, Takumi Kamiyama, Yasushi Izumi, Qingyin Qian, Yuma Yoshihashi, Yousuke Degawa, Kaori Watanabe, Yukako Hattori, Tadashi Uemura, Ryusuke Niwa

**Affiliations:** ^1^ Degree Programs in Life and Earth Sciences, Graduate School of Science and Technology, University of Tsukuba, Tennodai 1-1-1, Tsukuba, Ibaraki 305-8572, Japan; ^2^ Life Science Center for Survival Dynamics, Tsukuba Advanced Research Alliance (TARA), University of Tsukuba, Tennodai 1-1-1, Tsukuba, Ibaraki 305-8577, Japan; ^3^ Division of Cell Structure, National Institute for Physiological Sciences, Okazaki, Aichi 444-8787, Japan; ^4^ Department of Physiological Sciences, School of Life Science, SOKENDAI (Graduate University for Advanced Studies), Okazaki, Aichi 444-8585, Japan; ^5^ PhD Program in Human Biology, School of Integrative and Global Majors, University of Tsukuba, Tennodai 1-1-1, Tsukuba, Ibaraki 305-8577, Japan; ^6^ Sugadaira Research Station, Mountain Science Center, University of Tsukuba, Sugadairakogen 1278-294, Nagano 386-2204, Japan; ^7^ Graduate School of Biostudies, Kyoto University, Kyoto 606-8501, Japan; ^8^ Research Center for Dynamic Living Systems, Kyoto University, Kyoto 606-8501, Japan; ^9^ AMED-CREST, AMED, Otemachi 1-7-1, Chiyoda-ku, Tokyo 100-0004, Japan

**Keywords:** Generalist, Specialist, Gut epithelium, Immune system, Gut bacteria, Scopoletin

## Abstract

Organisms can generally be divided into two nutritional groups: generalists that consume various types of food and specialists that consume specific types of food. However, it remains unclear how specialists adapt to only limited nutritional conditions in nature. In this study, we addressed this question by focusing on *Drosophila* fruit flies. The generalist *Drosophila melanogaster* can consume a wide variety of foods that contain high glucose levels. In contrast, the specialist *Drosophila sechellia* consumes only the Indian mulberry, known as noni (*Morinda citrifolia*), which contains relatively little glucose. We showed that the lifespan of *D. sechellia* was significantly shortened under a high-glucose diet, but this effect was not observed for *D. melanogaster*. In *D. sechellia*, a high-glucose diet induced disorganization of the gut epithelia and visceral muscles, which was associated with abnormal digestion and constipation. RNA-sequencing analysis revealed that many immune-responsive genes were suppressed in the gut of *D. sechellia* fed a high-glucose diet compared with those fed a control diet. Consistent with this difference in the expression of immune-responsive genes, high glucose-induced phenotypes were restored by the addition of tetracycline or scopoletin, a major nutritional component of noni, each of which suppresses gut bacterial growth. We propose that, in *D. sechellia*, a high-glucose diet impairs gut immune function, which leads to a change in gut microbiota, disorganization of the gut epithelial structure and a shortened lifespan.

## Teaser

**Summary:** In the specialist fruit fly *Drosophila sechellia*, a high-glucose diet impairs gut immune function, leading to a change in gut microbiota and the gut epithelial disorganization structure, and a shortened lifespan.

## INTRODUCTION

In nature, there are two types of species regarding the type of food resources they rely on for survival: generalists, which use many resources, and specialists, which are limited to specific resources (Li et al., 2014; Loxdale et al., 2011). Generalists have been shown to have larger niches and geographic ranges than specialists. As specialist species use specific resources, their habitats are more heterogeneous and patchier than those of generalist species (Slatyer et al., 2013). Furthermore, nutritional ecology studies have shown that, because of their narrow variety of food choices, specialists can regulate nutrient balance only by controlling food intake, not by seeking balanced diets (Behmer, 2009; Poissonnier et al., 2018; Raubenheimer and Simpson, 2003). Therefore, specialists are more likely to be affected by nutritional imbalance than generalists. However, it remains unclear how specialists adapt to the limited nutritional conditions that are specific for survival.

Fruit flies belonging to the genus *Drosophila* include both generalist and specialist species; however, the two types of *Drosophila* species are thought to have evolved from a common ancestor (Anholt, 2020; Saisawang and Ketterman, 2014). For example, *D. melanogaster* and *D. simulans* are generalists that consume various foods (Markow, 2015; Markow and O'Grady, 2008), whereas *D. sechellia* is a specialist that is geographically restricted to the Seychelles Islands; however, all three species belong to the same taxonomic clade, the *melanogaster* species subgroup. *Drosophila sechellia* only consumes the Indian mulberry *Morinda citrifolia*, commonly known as noni (Salazar-Jaramillo and Wertheim, 2021), which contains few carbohydrates (Watanabe et al., 2019) (Fig. S1). Such differential food choice between *D. sechellia* and other species of the *melanogaster* subgroup is reflected by differences in their detoxification ability and chemoattraction characteristics. For example, noni is toxic to *D. melanogaster* and *D. simulans* (Jones, 2001), whereas *D. sechellia* is resistant to the toxins, partly because of the presence of the detoxifying gut bacteria *Lactiplantibacillus* (Heys et al., 2019 preprint). In addition, *D. melanogaster* and *D. simulans* are repelled by the octanoic and *n*-caproic acids present in noni, whereas *D. sechellia* is attracted to these fatty acids (Auer et al., 2021; Higa and Fuyama, 1993; Lanno et al., 2017; López et al., 2017; Matsuo et al., 2007; Prieto-Godino et al., 2017; Salazar-Jaramillo and Wertheim, 2021). However, when *D. melanogaster* ingests gut microbes that are usually harbored in *D. sechellia* gut, *D. melanogaster* is attracted to octanoic acid (Heys et al., 2021). Therefore, it is hypothesized that *D. sechellia* and its gut microbes may be evolutionarily and ecologically specialized to noni, allowing *D. sechellia* to avoid interspecific competition and achieve reproductive success.

Previous studies have investigated how the generalist *Drosophila* species and the specialist *D. sechellia* adapt to different nutritional conditions. For example, *D. sechellia* larvae cannot survive in sugar-rich conditions, while the generalist *D. simulans* is sugar tolerant (Melvin et al., 2018). When larvae of generalist and specialist species of *Drosophila* were fed diets with different carbohydrate-to-protein ratios, the diet with a higher carbohydrate-to-protein ratio decreased the survival rate of the specialist *D. sechellia* during larval development (Watanabe et al., 2019). Moreover, when adults of the two *Drosophila* species types were fed these diets after eclosion, the diet with a higher carbohydrate-to-protein ratio resulted in reduced egg production and a shortened lifespan in *D. sechellia*, but not in *D. melanogaster* (Watada et al., 2020).

The differences in the effects of the distinct diets can be partly explained by the differences in the carbohydrate-responsive pathways between the generalist *Drosophila* species and the specialist *D. sechellia*. For example, a study by Melvin et al. (2018) demonstrated that the genes involved in mitochondrial ribosome biogenesis and intracellular signaling, such as *PPP1R15/Gadd34* and *SERCA*, contribute to the sugar tolerance of *D. simulans*. Other studies showed that *D. melanogaster* has active carbohydrate-responsive pathways, including the TGF-β/activin signaling pathway (Chng et al., 2014, 2017; Ghosh and O'Connor, 2014; Mattila and Hietakangas, 2017; Mattila et al., 2015; Watanabe et al., 2019). In contrast, the noni-consuming *D. sechellia* lost those types of mechanisms during evolution and has become hypersensitive to a carbohydrate-rich diet (Melvin et al., 2018; Watanabe et al., 2019). However, the physiological mechanism behind high-carbohydrate adaptation has only been validated in larvae and has not been elucidated in adults. Thus, in this study, we aimed to investigate the effect of a high-glucose diet on *D. melanogaster* and *D. sechellia* adults and examine the mechanistic differences in high-glucose tolerance between these two closely related *Drosophila* species.

## MATERIALS AND METHODS

### Fly husbandry

The wild-type *Drosophila melanogaster* Meigen 1830 strain Oregon R, which has been maintained in R.N.’s lab for over 14 years, was used in this study. Wild-type *Drosophila sechellia* Tsacas and Baechli 1981 strain K-S10 was obtained from KYORIN-Fly (Fly Stocks of Kyorin University, Japan). *Drosophila melanogaster* and *D. sechellia* were both reared on a standard diet (see below) at 25°C with a light cycle of 12 h light and 12 h dark. In this study, we used virgin females for all assays.

### Fly diets

The control diet (CD) stock was a mixture of 100 ml distilled water, 10 g glucose, 9 g cornmeal, 4 g dry yeast, 1 g agar and 300 μl propionic acid, for a glucose concentration of 8% (w/w). The high-glucose diet (HGD) stock was a mixture of 100 ml distilled water, 50 g glucose, 9 g cornmeal, 4 g dry yeast, 1 g agar and 300 μl propionic acid, leading to 30% (w/w) glucose. To prepare tetracycline-supplemented diet, 200 mg of tetracycline (T3383, Sigma-Aldrich, St Louis, MO, USA) was added to 100 g of CD or HGD. We also prepared a stock of scopoletin-supplemented diet, which consisted of 100 g of CD or HGD with 11.7 mg scopoletin (S0367, Tokyo Kasei Kogyo Co. Ltd, Tokyo, Japan). In our experimental conditions, the volume of 100 g of CD or HGD was approximately 430 ml, leading to a scopoletin concentration of approximately 27.2 μg ml^−1^ in the food. This concentration was comparable to that of noni fruit juice (0.88–34.01 μg ml^−1^) reported previously (Deng et al., 2010). All diet preparations were stored at 4°C.

### *Drosophila* lifespan assay

To measure lifespan, a total of 10–20 unmated adult females were reared in 21 ml mini-vials (58.487, Sarstedt, Nümbrecht, Germany) containing approximately 3 g of food. The food was replenished every 3 days.

### Measurement of triacylglycerol

Five unmated females from each group were collected on the fifth day after eclosion. The flies were homogenized in 500 μl of phosphate-buffered saline (PBS) with 0.2% Triton X-100 using a BioMasher (Nippi, Yokohama, Japan), and were then incubated in a heat block at 70°C for 10 min. The samples were centrifuged at 17,800 ***g*** for 10 min at 4°C, and the supernatant was collected; 10 μl of the supernatant was used for protein quantification via the Bradford assay with a Coomassie Brilliant Blue protein assay solution (29449-15, Nacalai Tesque, Kyoto, Japan). The amount of triacylglycerol (TAG) in the whole body was measured in 10 μl of supernatant using a serum triglyceride measurement kit (TR0100, Sigma-Aldrich). The amount of free glycerol was subtracted from the measured value, and the subtracted value was normalized to the amount of protein.

### Measurement of circulating glucose level

We extracted the body fluids from *Drosophila* adults to measure the concentration of glucose. The thoraxes of 30–40 adult females (5–6 days after eclosion) were punctured with a tungsten needle, placed in 1.5 ml tubes, and centrifuged at 9000 ***g*** for 10 min to collect the body fluids. The body fluids (1 μl) were mixed with 99 μl of trehalase buffer (5 mmol l^−1^ Tris-HCl, 137 mmol l^−1^ NaCl, 2.7 mmol l^−1^ KCl pH 6.6) and the samples were incubated at 70°C for 5 min. The glucose level of the body fluids was measured by testing 30 μl of the resulting supernatant with a Glucose Assay Kit (GAGO20-1KT, Sigma-Aldrich).

### Evaluation of stone formation in the Malpighian tubules

To determine the level of stone formation in the Malpighian tubules, we observed the tubules of unmated females reared on CD or HGD at 5 days post-eclosion. The Malpighian tubules were dissected in PBS. The level of stone formation in the Malpighian tubules was evaluated on a five-point scale (0–4), as previously described (van Dam et al., 2020). To evaluate the level of stone formation under each dietary treatment, we calculated the average score for each treatment group.

### Measurement of intestinal alkaline phosphatase activity

To investigate the barrier function of the gut, we examined the activity of intestinal alkaline phosphatase (IAP), an intestinal mucosal defense factor that influences intestinal permeability, as previously described (Pereira et al., 2018). To measure IAP activity, we used para-nitrophenyl phosphate (pNPP; P0757S, New England Biolabs, Ipswich, MA, USA), a general phosphatase chromogenic substrate, as previously described (Pereira et al., 2018). The guts of approximately five unmated females at 5 days post-eclosion were dissected in PBS and homogenized in 160 µl of reaction solution (25 mmol l^−1^ sodium acetate pH 5.0, 10 mmol l^−1^ pNPP, 1 mmol l^−1^ DTT, 20% glycerol) with a protease inhibitor cocktail (Complete Mini EDTA-free tablets, 11836170001, Roche, Basel, Switzerland). The homogenates were thoroughly mixed and incubated at 30°C for 50 min. To stop the reaction, 125 µl of 0.32 mol l^−1^ NaOH was added to 75 µl of the reaction solution, and its absorbance was measured at 405 nm (Niwa et al., 2002). To measure the amount of protein in each sample, 125 μl of Coomassie Brilliant Blue protein assay solution was added to 75 μl of the reaction solution, and the absorbance was measured at 595 nm. The absorbance at 405 nm was normalized according to the protein content.

### Feeding experiment with blue dye

Blue dye (Erioglaucine, 861146, Sigma-Aldrich) was mixed with the diet to a concentration of 0.16%. Flies were allowed to feed on the diet with blue dye for 24 h. Then, they were homogenized in 200 µl PBS, and the number of adults used for homogenization was noted. The homogenate was centrifuged at 17,800 ***g*** for 10 min and 90 µl of the supernatant was dispensed into each well of a 96-well plate. The absorbance at 625 nm was measured and the obtained value was normalized according to the number of guts used.

### Calcofluor White staining

The guts of unmated females at 5 days post-eclosion were dissected in 50 mmol l^−1^ Tris-HCl. The dissected guts were placed on a glass slide, 100 μl of Calcofluor White stain (18909, Sigma-Aldrich) was placed onto the tissue and 100 μl of 10% KOH solution was added. The glass slide was lightly shaken to mix the solutions and a glass coverslip was then placed on the slide. The samples stained with Calcofluor White were observed under UV light at λ_ex_=355 nm.

### Counting of feces

Newly eclosed, unmated females were reared under CD or HGD conditions for 5 days. Twenty flies were then placed in empty vials without any food for 4 h, and the flies originally reared on CD or HGD were left to feed on the same food stained with blue dye (Erioglaucine; final concentration of 0.16%) for 3 h. The flies were then transferred into new empty vials without any food, and the number of feces droplets on the vial walls was counted.

### Immunohistochemistry

Unmated females at 5 days post-eclosion were dissected in PBS. The dissected guts were fixed in 4% paraformaldehyde/PBS for 1 h and the tissue was washed 3 times with PBT (PBS with 0.1% Triton X-100). After washing, the tissues were rinsed with a graded series of ethanol solutions (10%, 30% and 70% ethanol), and then further dehydrated with 100% ethanol for 15 min. The dehydrated guts were washed 3 times with PBT and blocked with blocking solution [2% bovine serum albumin (BSA)/PBT] for 1 h at room temperature. The blocked tissues were treated with the following primary antibodies diluted in blocking solution and incubated at 4°C overnight: mouse anti-Coracle (Cora) antibody (1:100, Developmental Studies Hybridoma Bank, DSHB C615.16), mouse anti-Discs large (Dlg) antibody (1:50; DSHB 4F3), anti-Mesh antibody (1:1000) (Izumi et al., 2016), rabbit anti-Phospho-Ezrin [Thr567]/Radixin [Thr564]/Moesin [Thr558] (pEzrin) antibody 48G2 (1:200; 3726S, Cell Signaling Technology, Danvers, MA, USA), rabbit anti-tachykinin (Tk) antibody (1:50; a gift from Jan Veenstra) (Veenstra et al., 2008) and anti-Tetraspamin-2A (Tsp2A) antibody (1:1000) (Izumi et al., 2016). After primary antibody treatment, the tissues were washed with PBT. The samples were then incubated with a blocking solution containing goat anti-mouse IgG conjugated with Alexa Fluor 488 (1:200; A32723, Thermo Fisher Scientific, Waltham, MA, USA) or goat anti-rabbit IgG conjugated with Alexa Fluor 555 (1:200; A32732, Thermo Fisher Scientific) and phalloidin conjugated with Alexa Fluor 546 (1:200; A22283, Thermo Fisher Scientific) under light-shielded conditions for 2 h at room temperature. The tissues were then washed with PBT for 30 min, with nuclear staining with 4′,6-diamidino-2-phenylindole (DAPI; 1:1000, diluted in PBT) performed for 15 min. FluorSave reagent (345789, Merck Millipore, Burlington, MA, USA) was used for mounting the samples on glass slides.

### Electron microscopy

Unmated females at 5 days post-eclosion were dissected in ultrapure water (Milli-Q; Sigma-Aldrich). The dissected guts were fixed in a mixture of 2% paraformaldehyde, 2.5% glutaraldehyde and 0.1 mol l^−1^ cacodylate (pH 7.4) for 1 h at room temperature. After fixation, the guts were washed in 0.1 mol l^−1^ cacodylate buffer and post-fixed in 1% osmium tetroxide with 0.1 mol l^−1^ cacodylate buffer (pH 7.4) for 1 h at room temperature. The guts were washed with distilled water and stained with 0.5% uranyl acetate for 2 h at room temperature. After three washes with distilled water, the guts were dehydrated in a graded series of ethanol solutions (65%, 75%, 85%, 95% and 99.5%) and transferred to 100% ethanol. The guts were then soaked in propylene oxide, transferred to a 1:1 mixture of propylene oxide and Quetol 812 resin (Nisshin-EM, Tokyo, Japan), and embedded in Epon 812 resin. Ultrathin sections of approximately 60 nm thickness were collected on copper grids, stained with 0.5% uranyl acetate and then stained with a lead solution containing 1% lead citrate, 1% lead nitrate and 2% sodium citrate (Sato, 1968). The sections were washed with distilled water and dried. The sections were observed using a JEM-1010 electron microscope (JEOL, Tokyo, Japan) equipped with a Veleta TEM CCD camera (Olympus, Tokyo, Japan) at an accelerating voltage of 80 kV.

### RNA-sequencing and gene ontology analysis

RNA sequencing (RNA-seq) was performed on unmated females of both species reared on CD or HGD at 5 days post-eclosion to analyze the genes whose expression was altered on HGD compared with CD in each species. Total RNA was extracted from 30 unmated females for each species in each condition using RNAiso Plus (9101, TaKaRa Bio, Kusatsu, Shiga, Japan) and an RNeasy Mini kit (74104, Qiagen, Hilden, Germany). We had three biological replicates for each species in each condition. An average of 20 million reads was sequenced for each biological replicate. For quantification of gene expression, FASTQ files containing the raw sequence reads were assessed for quality using FASTQC. The sequences were trimmed at 1 nucleotide from the 3′ end and at the adaptor sequences, and reads with a length of <20 nucleotides were trimmed from the raw single-end reads using Trim Galore 0.6.4 (Babraham Bioinformatics, Cambridge, UK). Reads were mapped using HISAT2 (version 2.1.0) (Kim et al., 2019) to the BDGP *D. melanogaster* genome (dm6) downloaded from FlyBase (Larkin et al., 2021) or the *D. sechellia* genome (ASM438219v1) from the datasets of the National Center for Biotechnology Information (NCBI). The gtf files (dmel-all-r6.30.gtf) were downloaded from FlyBase for *D. melanogaster* and the NCBI database (release 101) for *D. sechellia*. Samtools (version 1.9) (Li et al., 2009) and Stringtie (version 2.0.6) (Pertea et al., 2016) were used to sort, merge and count the number of reads mapped to each gene. The number of trimmed mean of M-values-normalized fragments per kilobase of combined exon length per one million of total mapped reads (TMM-normalized FPKM value) was calculated, and differential expression analysis was performed using R (version 3.6.1), Ballgown (version 2.18.0) (Pertea et al., 2016) and edgeR (version 3.28.0) (McCarthy et al., 2012; Robinson et al., 2010). Genes with a Benjamini–Hochberg false discovery rate (FDR)<0.01 were identified as differentially expressed genes (DEGs). *Z*-score was calculated using python (version 3.8.8) and scipy (version 1.6.2).

*Drosophila melanogaster* orthologs of each *D. sechellia* gene were downloaded from FlyBase (dmel_orthologs_in_drosophila_species_fb_2021_03.tsv). If a *D. sechellia* gene was not orthologous to any *D. melanogaster* gene annotated by FlyBase, we manually searched for *D. melanogaster* ortholog(s) using the NCBI database and assigned any orthologous relationships we found. DEGs of *D. sechellia*, and *D. melanogaster* orthologs of *D. sechellia* DEGs were uploaded to Metascape (Zhou et al., 2019) to conduct gene ontology (GO) enrichment analyses and calculate *P*-values (see figure legends).

### Colony formation assay

A colony formation assay was conducted to determine the amount of gut bacteria present in adult *Drosophila*. Five bacterial culture media were used: brain heart infusion (BHI) broth [18.5 g Bacto BHI (Becton Dickinson 237500), 7.5 g agar and 500 ml distilled water]; lysogeny broth [LB; 10 LB tablets with agar (Lennox, Sigma-Aldrich L7025) and 483 ml distilled water]; de Man, Rogosa and Sharpe (MRS) broth [26 g MRS broth (Oxoid CM0359), 7.5 g agar and 500 ml distilled water]; liver infusion broth [LIB; Difco LIB (Becton Dickinson 226920), 7.5 g agar and 500 ml distilled water]; and mannitol [12.5 g d-Mannitol (Sigma-Aldrich M4125), 1.5 g Bacto Peptone (Beckton Dickinson 211677), 2.5 g select yeast extract (Sigma-Aldrich Y1000), 7.5 g agar and 500 ml distilled water]. The guts of 10 HGD-treated unmated females at 5 days post-eclosion were dissected in 50 mmol l^−1^ Tris-HCl. The dissected guts were placed in 250 µl of each liquid medium, and the tissues were mashed using a BioMasher (Nippi). The gut sample solutions were diluted 1–1/16. After 5 days of incubation, the number of colonies growing on each plate was counted, and the colony-forming units (CFU) were calculated. The number of replicates used is given in the individual figure legends.

### Visualization of lipid droplets in the gut

Staining was performed using LipidTOX, as previously described (Bailey et al., 2015). Unmated females at 5 days post-eclosion were dissected in PBS. The dissected guts were fixed in 4% paraformaldehyde/PBS for 40 min. The fixed tissues were washed 3 times with PBS and 0.2% Triton X-100. After washing, the guts were stained with LipidTOX and DAPI (diluted 1:1000 in PBS and 0.2% Triton X-100) for 2 h under light-shielded conditions. FluorSave reagent (Merck Millipore) was used to mount the samples on glass slides.

## RESULTS

### *Drosophila sechellia* lifespan is shortened under high-glucose conditions

**Fig. 1.**
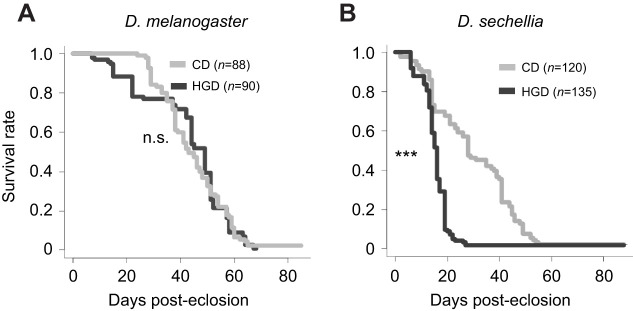
**A high-glucose diet induces a shortened lifespan in *Drosophila sechellia*.** Lifespan of (A) *D. melanogaster* and (B) *D. sechellia* reared on a control diet (CD) and high-glucose diet (HGD). The numbers of flies (*n*) used in the assays is indicated. ***P<0.0001 (log-rank test). n.s., not significant.

First, we examined the effect of a HGD on the adult lifespan of *D. melanogaster* and *D. sechellia*. In all assays, we used virgin females to exclude the possibility of species-specific contributions of egg laying to lifespan (Watada et al., 2020). We raised wild-type strains of these *Drosophila* species from the larval to pupal stage on a CD with 8% (w/w) glucose. We then allocated the newly eclosed adult flies into the CD or HGD treatment groups, the latter of which was prepared by adding excess glucose to the CD, leading to 30% (w/w) glucose. This methodology was chosen because several previous studies have utilized a 30% glucose diet (May et al., 2019; Musselman and Kühnlein, 2018; Musselman et al., 2011; Na et al., 2013). We found no change in the lifespan of *D. melanogaster* between the CD and HGD groups (Fig. 1A). In contrast, HGD drastically shortened the lifespan of *D. sechellia* compared with CD (Fig. 1B), suggesting that *D. melanogaster* and *D. sechellia* are tolerant and sensitive to diets with a high glucose content, respectively.

### Food intake, TAG and blood glucose levels, and tubular stone formation are not associated with the shortened lifespan of *D. sechellia* reared on HGD

We next investigated how behavioral and physiological responses to HGD differed between *D. melanogaster* and *D. sechellia*. We found no differences in food intake between these species (Fig. S2A), suggesting that excessive glucose intake does not account for the shortened lifespan induced by HGD in *D. sechellia*.

It is well known that high-sugar diets result in increased TAG levels, blood glucose levels and stone formation in the Malpighian tubules of *D. melanogaster*, and these factors are all associated with a shortened lifespan (Hofbauer et al., 2021; Liao et al., 2021; van Dam et al., 2020). Therefore, we examined whether these phenotypes could be observed in *D. sechellia* under HGD conditions. We found that TAG levels and blood glucose concentrations were elevated in both *D. melanogaster* and *D. sechellia* under HGD conditions (Fig. S2B,C). In contrast, the level of Malpighian tubule stone formation was not enhanced but was suppressed in *D. sechellia* compared with *D. melanogaster* under HGD conditions (Fig. S2D). These results suggest that TAG levels, blood glucose levels and tubular stone formation are unlikely to be responsible for the shortened lifespan of *D. sechellia* under HGD conditions.

### Disrupted gut epithelial structure in *D. sechellia* reared on HGD

**Fig. 2.**
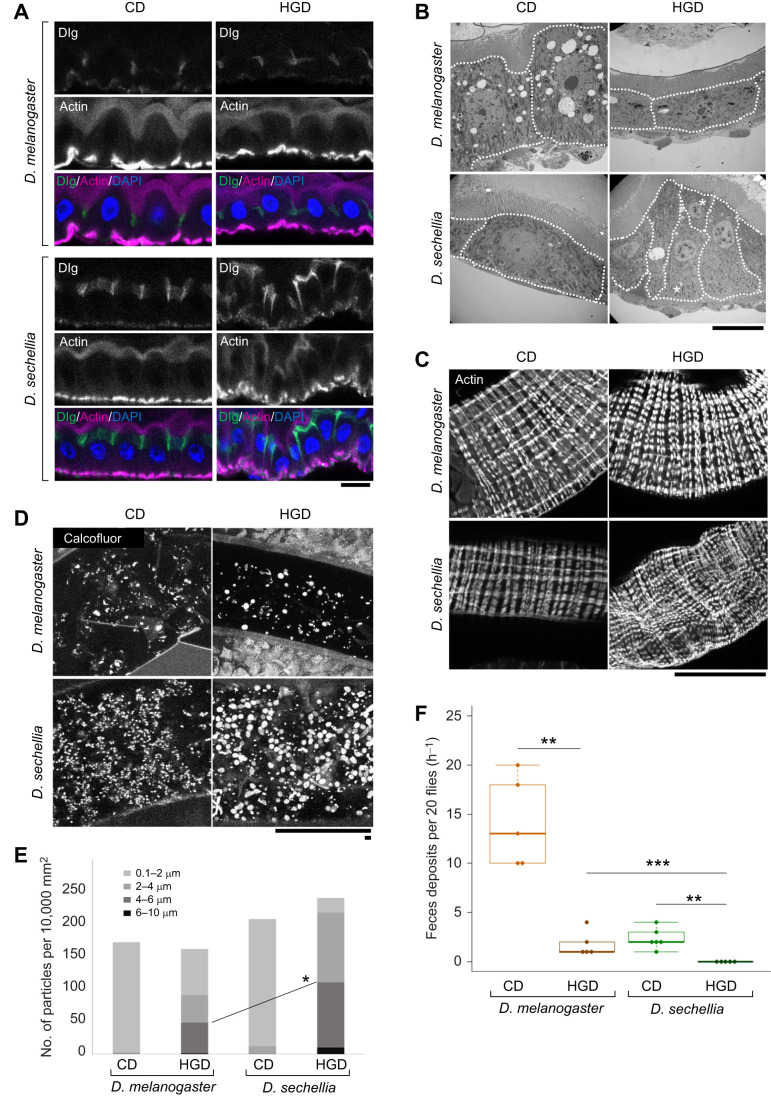
**A high-glucose diet induces gut epithelial disorganization in *D. sechellia*.** (A) Immunohistochemical observation of the gut epithelial structures of *D. melanogaster* and *D. sechellia* reared on CD and HGD. The gut epithelia were stained with fluorescent phalloidin (magenta), anti-Discs large (Dlg; green) and DAPI (blue), which were used to visualize actin filaments, septate junctions and nuclei, respectively. We focused on the R2 region for our observations. The upper and lower parts of each image correspond to the apical and basal sides of the gut epithelium, respectively. Scale bar: 10 μm. (B) Electron microscopy of the gut epithelial structures of *D. melanogaster* and *D. sechellia*. The upper and lower parts of each image correspond to the apical and basal sides of the gut epithelium, respectively. Asterisks indicate two cells that were aligned along the apicobasal axis. Scale bar: 10 μm. (C) Intestinal muscle fibers surrounding the gut epithelia visualized by fluorescent phalloidin. Scale bar: 100 μm. (D) Calcofluor White staining for visualization of bacteria, fungi and dietary dry yeast cell particles. Long and short scale bars: 100 and 5 μm, respectively. (E) Distribution of different-sized particles in the *D. melanogaster* and *D. sechellia* gut. Note that, in HGD conditions, the large particles (diameter of more than 4–5 μm), which mainly corresponded to dietary dry yeast, were increased in the gut of *D. sechellia* as compared with that of *D. melanogaster*. The difference in the ratio of particles >4 μm between *D. melanogaster* and *D. sechellia* reared on HGD was statistically significant (*P<0.05, χ^2^-test). (F) Numbers of feces deposited in 1 h from 20 adult female flies of *D. melanogaster* and *D. sechellia* (*n*=5 each). Box plots show median values, upper and lower quartiles, 1.5× interquartile range and outliers. Notably, no feces were observed from *D. sechellia* reared on HGD in five independent experiments. **P<0.001, ***P<0.0001 (Student's *t*-test with Bonferroni's correction).

Previous studies have shown that intestinal structure and the gut environment, which includes proper maintenance of the gut epithelium, gut immune system and gut microbiota, affect the lifespan of *D. melanogaster* (Biagi et al., 2016; Biteau et al., 2010; Boehme et al., 2021; Claesson et al., 2012; Guo et al., 2014; Keebaugh et al., 2018; Li et al., 2016; Loch et al., 2017; Mackowiak, 2013). However, the relationship between gut function and lifespan in *D. sechellia* has not been examined. Therefore, we examined whether and how the intestinal structure and environment of *D. sechellia* were altered under HGD conditions. First, we visualized the nuclei, actin cytoskeleton, apical surface and septate junctions of the gut epithelial structure. There were no visible changes in the cell morphology or sheet structure of the gut epithelia of *D. melanogaster* between the CD and HGD conditions, as these insects exhibited uniform monolayer epithelia (Fig. 2A; Fig. S3A). In contrast, disorganization of gut epithelia occurred in *D. sechellia* under HGD but not CD conditions (Fig. 2A; Fig. S3A). Specifically, under HGD conditions, *D. sechellia* gut epithelia showed frequent undulation (Fig. 2A), and the posterior midgut exhibited a higher frequency of disorganization than the anterior midgut. Furthermore, electron microscopy revealed that two cells were often aligned along the apicobasal axis of the epithelium (Fig. 2B, asterisks). These abnormalities were not observed in *D. melanogaster* under either CD or HGD conditions or in *D. sechellia* under CD conditions (Fig. 2A,B).

We also found that the distribution of septate junction proteins (Dlg, Tsp2A, Mesh and Cora) and an apical marker protein (pEzrin) was altered in *D. sechellia* reared on HGD, probably as a result of intestinal epithelial disorganization (Fig. 2A; Fig. S3A). Nevertheless, the apicobasal polarity of the *D. sechellia* gut epithelium under HGD conditions was not severely affected, as Dlg, Dsp2A, Mesh and Cora were still localized in the basolateral region, and pEzrin was localized at the apical surface (Fig. 2A; Fig. S3A). These results suggest that, in *D. sechellia*, HGD affects gut epithelial morphology independently of apicobasal polarity*.*

We also investigated whether the intestinal muscle fibers surrounding the gut epithelia were affected by a high-glucose diet. In *D. melanogaster*, there were no changes in the myofiber structure of the two types of visceral muscle (i.e. circular and vertical visceral muscles) between CD and HGD conditions (Fig. 2C). In contrast, in the gut of *D. sechellia* reared on HGD, disorganized myofibers were observed in both types of visceral muscle (Fig. 2C). Based on these results, we speculate that HGD-induced disorganization of the gut epithelia and visceral muscles might be responsible for the shortened lifespan of *D. sechellia*.

As gut barrier dysfunction is frequently associated with a shortened lifespan (Clark et al., 2015; Pereira et al., 2018; Rera et al., 2012), we evaluated whether gut barrier function was impaired in *D. sechellia* reared on the HGD. For this purpose, we measured the activity of IAP, an intestinal mucosal defense factor that influences gut permeability (Pereira et al., 2018). While IAP activity in *D. melanogaster* gut remained unchanged under CD and HGD conditions, the *D. sechellia* gut under HGD conditions exhibited higher IAP activity than under CD conditions (Fig. S2E). These results imply that the gut barrier function in *D. sechellia* is also affected to some extent under HGD conditions.

### Diet-derived dry yeast cell particles accumulate in the gut lumen of *D. sechellia* reared on HGD

Previous studies have reported that the deformation of muscle fibers surrounding the gut may disrupt gut peristalsis, impairing the digestion and absorption of ingested food (Aghajanian et al., 2016; Schröter et al., 2006). Therefore, we expected that the disrupted epithelial structure of the gut might influence the enteral contents in the gut lumen of *D. sechellia* reared under HGD conditions. This expectation was supported by our staining experiment with Calcofluor White, which binds to cellulose and chitin in bacterial and fungal cell walls (Monheit et al., 1984). We realized that Calcofluor White could also be used to visualize dry yeast cell particles (5–6 μm diameter), which were used to prepare the *Drosophila* diets (Fig. S3B). In the gut lumen of *D. sechellia* fed with HGD, aberrant enrichment of 5–6 μm diameter particles stained with Calcofluor White was observed (Fig. 2D). In contrast, 5–6 μm diameter particles were rarely observed in *D. melanogaster* under either CD or HGD conditions or in *D. sechellia* under CD conditions. However, there were smaller (0.1–4 μm) particles stained with Calcofluor White for these treatments, which probably corresponded to bacteria or remnants of digested dry yeast in the lumen (Fig. 2D). We confirmed that most of the large particles were dry yeast cell particles, as there were remarkably fewer 5–6 μm diameter particles in the gut lumen of *D. sechellia* reared on a diet without dry yeast (Fig. S3C). Moreover, the amount of feces was significantly reduced in both species under HGD conditions, with no feces observed for *D. sechellia* raised on HGD (Fig. 2F). These results imply that the disorganization of the gut epithelia and visceral muscles of *D. sechellia* under HGD conditions leads to the abnormal accumulation of consumed food in the gut lumen owing to impaired gut digestive function.

### Expression of genes activating gut immune function is downregulated in *D. sechellia* reared on HGD

**Fig. 3.**
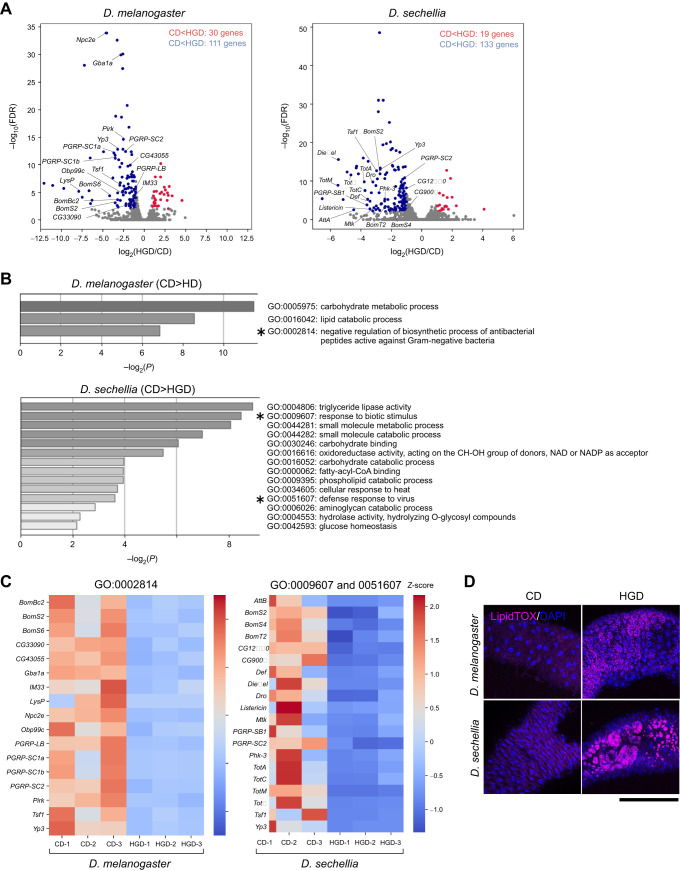
**Differences in the transcriptome and lipid droplet formation of *D. melanogaster* and *D. sechellia* on the two diets.** (A) Volcano plots representing changes in gene expression for flies fed CD and HGD. The annotated gene sets of *D. melanogaster* and *D. sechellia* used in this study were defined based on the FlyBase and NCBI datasets (see also Tables S1 and S2). The *x*-axis represents binary logarithmic values (log_2_) of the fold-change (FC) of gene expression (i.e. expression level of each gene under HGD conditions divided by that under CD conditions). The *y*-axis represents logarithmic values (log_10_) of the false discovery rate (FDR). Blue dots represent differentially expressed genes (DEGs) with log_2_FC<−1 and FDR<0.01. Red dots represent DEGs with log_2_FC>1 and FDR<0.01. Names of representative immune-related genes classified into the following gene ontology (GO; shown in B) are displayed: GO:0002814 (negative regulation of biosynthetic process of antibacterial peptides active against Gram-negative bacteria) in *D. melanogaster*, and GO:0009607 (response to biotic stimulus) and GO:0051607 (defense response to virus) in *D. sechellia*. (B) Significantly enriched GO terms of the DEGs (shown in blue in A). GO terms for each *D. sechellia* gene were assigned based on the information of a *D. melanogaster* ortholog (see Table S2). The GO terms with corrected *P*-values <0.01 were considered significantly enriched by DEGs. The intensity of the gray bars varies depending on whether the range of values of −log_10_(*P*) was 2–4, 4–6, 6–8 or 8–10. Asterisks represent GO classifications that include immune-responsive genes. (C) Expression heatmap of a curated set of DEGs classified into GO:0002814 in *D. melanogaster*, and GO:0009607 and GO:0051607 in *D. sechellia*. Note that all the genes of GO:0051607 are included in GO:0009607. Gene expression levels are based on TMM-normalized FPKM. Three replicates for each experimental condition are represented. Red and blue indicate increased and decreased gene expression relative to average gene expression levels of all six samples of *D. melanogaster* and *D. sechellia*, respectively; the *Z*-scores are plotted on a color scale (right). *Att*, *attacin*; *Bom*, *bomamin*; *Def*, *defensin*; *Dro*, *drosomycin*; *Gba*, *glucocerebrosidase*; *IM*, *immune induced molecule*; *Lys*, *lysozyme*; *Mtk*, *metchnikowin*; *Obp*, *odorant-binding protein*; *PGRP*, *peptidoglycan recognition protein*; *Phk*, *pherokine*; *Pirk*, *poor Imd response upon knock-in*; *Tot*, *Turandot*; *Tsf*, *transferrin*; *Yp*, *yolk protein*. (D) Lipid droplets visualized by LipidTOX in the gut epithelia of *D. melanogaster* and *D. sechellia*. Red: LipidTOX; blue, DAPI. Scale bar: 100 μm.

Next, we characterized the HGD-induced gut dysfunction in *D. sechellia* using transcriptomic analysis. We conducted an RNA-seq analysis of the gut of *D. melanogaster* and *D. sechellia* reared on CD and HGD. We performed differential expression analysis and compared gene expression between flies reared on CD and HGD in each species (see Materials and Methods). We then focused on genes whose expression levels were altered more than 2-fold in HGD versus CD conditions and whose FDR was <0.01 (Fig. 3A; Table S1). We then performed a GO analysis of the DEGs. We found that a certain number of downregulated genes were classified into GO terms related to carbohydrate and lipid metabolism (Fig. 3B). Therefore, the gene expression profile of such nutritional metabolism cannot account for the HGD-induced gut phenotypes of *D. sechellia.*

In contrast, one characteristic difference between the two species was the classification of GO terms related to immune function (Fig. 3A,B). In *D. melanogaster* reared under HGD conditions, the expression of genes classified as GO:0002814 was decreased (Fig. 3A–C; Table S1). GO:0002814 includes genes related to the ‘negative regulation of biosynthetic process of antibacterial peptides active against Gram-negative bacteria’, such as genes encoding Bomanin and peptidoglycan recognition proteins, suggesting that gut immune function might be enhanced in *D. melanogaster* under HGD conditions. In contrast, in *D. sechellia*, the expression of genes classified as GO:0009607 and GO:0051607 was significantly decreased under HGD conditions (Fig. 3A–C; Table S2). GO:0009607 and GO:0051607 include genes related to ‘response to biotic stimulus’ and ‘defense response to viruses’, respectively, such as genes encoding antimicrobial peptides (the Turandot family of proteins, Defensin, Drosocin, Metchnikowin and some immune-induced peptides) and genes related to the Toll pathway and the immune deficiency (IMD) pathway. These results suggest that, in contrast to *D. melanogaster*, *D. sechellia* gut immune function is impaired when reared on HGD.

A previous study reported that *D. melanogaster* with mutations in the IMD pathway had large lipid droplets in the gut epithelial cells as a result of an imbalance of the gut bacteria, and that the formation of these droplets was caused by the suppressed production of the peptide hormone tachykinin (Tk) in enteroendocrine cells (Kamareddine et al., 2018). Therefore, we examined whether intestinal lipid droplets were altered in *D. sechellia* under HGD conditions using LipidTOX staining. The lipid droplets were larger in the gut epithelium of *D. sechellia* reared under HGD conditions compared with those in *D. melanogaster* (Fig. 3D). However, the protein level of Tk in enteroendocrine cells did not change in either *D. melanogaster* or *D. sechellia* under CD or HGD conditions (Fig. S2F,G). Therefore, HGD-induced large lipid droplet formation in *D. sechellia* resembles IMD pathway-mediated lipid droplet formation in *D. melanogaster*, though it may be generated by a different mechanism from the Tk-dependent mechanism.

### Addition of tetracycline restores the shortened lifespan and disrupted gut epithelial structure in *D. sechellia* under HGD conditions

**Fig. 4.**
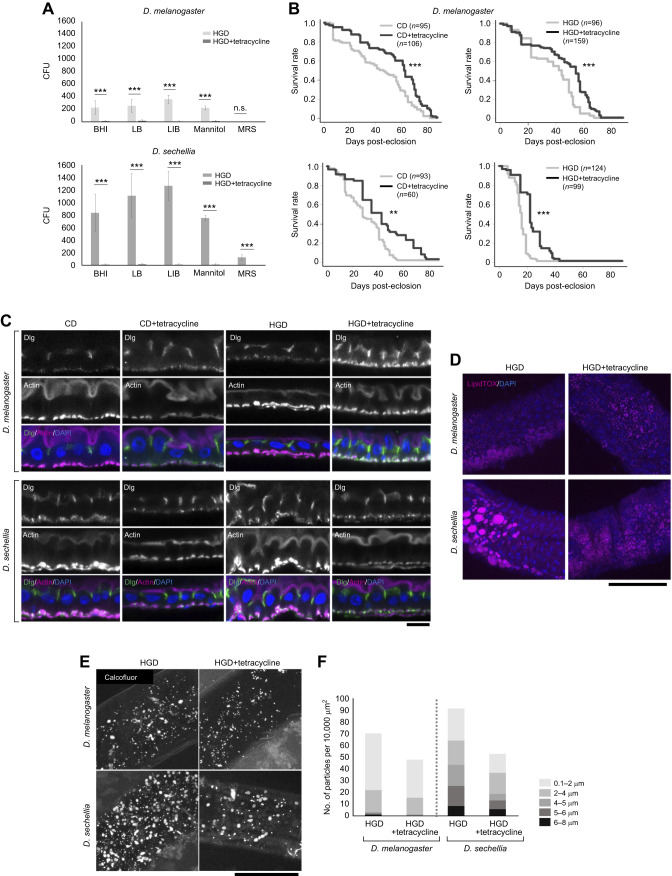
**HGD-induced phenotypes are recovered by tetracycline treatment.** (A) Colony formation assay using gut lysates derived from *D. melanogaster* and *D. sechellia* reared on HGD with or without tetracycline. The *x*-axis represents five types of bacteria culture medium: brain heart infusion (BHI), lysogeny broth (LB), liver infusion broth (LIB), mannitol, and de Man, Rogosa and Sharpe broth (MRS). The *y*-axis shows the number of colony forming units (CFU) from 10 plates of each bacterial culture media (means±s.e.m.). ***P<0.001 (Tukey–Kramer test). n.s., not significant. (B) Lifespan of *D. melanogaster* and *D. sechellia* reared on CD and HGD with or without tetracycline. The number of flies (*n*) used in the assays is indicated. **P<0.001 and ***P<0.0001 (log-rank test). (C) Immunohistochemical observation of the gut epithelial structures of *D. melanogaster* and *D. sechellia* reared on CD and HGD with or without tetracycline. The gut epithelia were stained with fluorescent phalloidin (magenta), anti-Discs large (Dlg; green) and DAPI (blue) to visualize the actin filaments, septate junctions and nuclei, respectively. We focused on the R2 region for our observations. The upper and lower parts of each image correspond to the apical and basal sides of the gut epithelium, respectively. Scale bar: 10 μm. (D) LipidTOX visualization of lipid droplets in the gut epithelium of *D. melanogaster* and *D. sechellia* reared on HGD with or without tetracycline. Red: LipidTOX; blue, DAPI. Scale bar: 100 μm. (E) Calcofluor White staining used to visualize bacteria, fungi and dietary dry yeast cell particles in HGD conditions with or without tetracycline. Long and short scale bars: 100 μm and 5 μm, respectively. (F) Distribution of differently sized particles in the *D. melanogaster* and *D. sechellia* gut in HGD conditions with or without tetracycline. Note that the large particles (diameter >4–5 μm), which mainly corresponded to dietary dry yeast, were reduced by tetracycline treatment in the gut of *D. sechellia*.

Considering that many positive regulators of immune responses have downregulated expression in the gut of *D. sechellia* reared on HGD, we hypothesized that some gut bacteria present in *D. sechellia* might be involved in shortening the lifespan of *D. sechellia* under HGD conditions. To test this hypothesis, we examined whether the shortened lifespan of *D. sechellia* reared on HGD was suppressed when the flies were fed tetracycline, an antimicrobial agent (Chopra and Roberts, 2001). A colony formation assay with five types of bacterial culture media (BHI, LB, LIB, MRS and mannitol media) confirmed that tetracycline treatment significantly suppressed bacterial growth in the gut of *D. melanogaster* and *D. sechellia* (Fig. 4A). We then measured the lifespan of both *Drosophila* species after being fed CD and HGD with the addition of tetracycline. We found that tetracycline treatment prolonged lifespan in both *D. melanogaster* and *D. sechellia* under CD and HGD conditions (Fig. 4B). Tetracycline treatment-induced longevity has already been reported in a previous study (Obata et al., 2018). These results suggest that some of the gut microbiota are involved in shortening the lifespan of *Drosophila*, irrespective of species or diet. Nevertheless, we emphasize that tetracycline treatment extended the lifespan of *D. sechellia* to some extent even under HGD conditions (Fig. 4B). Therefore, it is likely that the gut bacteria are partly responsible for the shortened lifespan of *D. sechellia* under HGD conditions.

We also observed the gut epithelial structures of *Drosophila* fed diets containing tetracycline. In *D. melanogaster*, there was no significant change in the gut epithelial structure with the addition of tetracycline (Fig. 4C). In contrast, tetracycline suppressed the disruption of the gut epithelial structure in *D. sechellia* under HGD conditions, as the gut epithelia under tetracycline treatment were seldom undulating (Fig. 4C). Additionally, in the *D. sechellia* gut, the size of the lipid droplets became smaller, and lipid accumulation was suppressed (Fig. 4D). In contrast, there was no dramatic change in the size of the lipid droplets in the gut of *D. melanogaster* fed HGD with tetracycline. Moreover, tetracycline treatment led to a reduction in the number of Calcofluor White-positive 5–6 μm particles that corresponded to dry yeast in the gut of *D. sechellia* under HGD conditions (Fig. 4E,F). These results suggest that gut bacteria are crucial for gut epithelial disorganization and food digestion in *D. sechellia* under HGD conditions.

### Addition of scopoletin restores the shortened lifespan and the disrupted gut epithelial structure in *D. sechellia* under HGD conditions

**Fig. 5.**
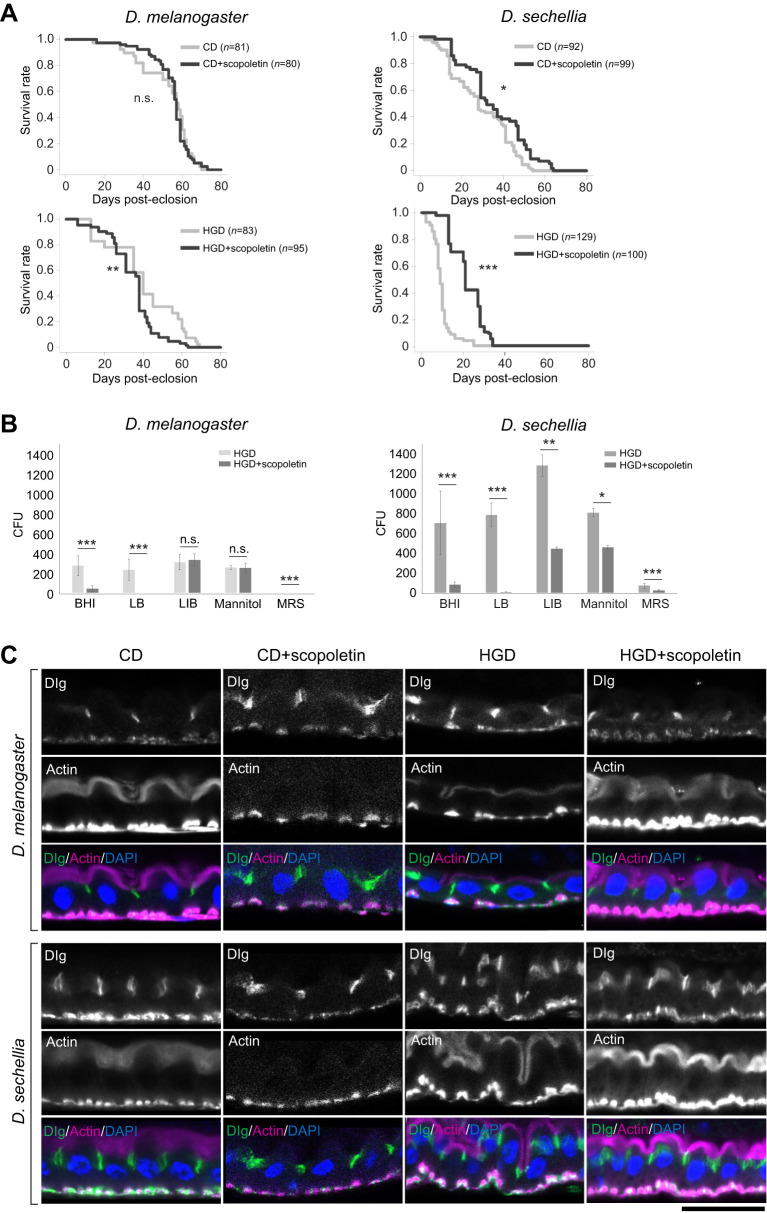
**HGD-induced abnormalities of *D. sechellia* are suppressed by scopoletin, a major nutrient in noni.** (A) Lifespan of *D. melanogaster* and *D. sechellia* reared on CD and HGD, with and without scopoletin. The number of flies (*n*) used in the assays is indicated. *P<0.05, **P<0.001 and ***P<0.0001 (log-rank test). n.s., not significant. (B) Colony formation assay using gut lysates derived from *D. melanogaster* and *D. sechellia* reared on HGD with or without scopoletin. The *x*-axis shows the five types of bacterial culture media. The *y*-axis shows the number of CFU from six plates of each bacterial culture medium (means±s.e.m.). *P<0.05, **P<0.01 and ***P<0.001 (Tukey–Kramer test). n.s., not significant. (C) Immunohistochemical observation of the gut epithelial structures of *D. melanogaster* and *D. sechellia* reared on CD and HGD, with or without scopoletin. Gut epithelia were stained with fluorescent phalloidin (magenta), anti-Discs large (Dlg; green) and DAPI (blue) to visualize actin filaments, septate junctions and nuclei, respectively. We focused on the R2 region for our observations. The upper and lower parts of each image correspond to the apical and basal sides of the gut epithelium, respectively. Scale bar: 10 μm.

*Drosophila sechellia* is a specialist that only consumes noni (Anholt, 2020; Saisawang and Ketterman, 2014). Therefore, from a nutritional perspective, we examined whether the dietary addition of a major nutrient present in noni would affect lifespan. Previous studies have reported that scopoletin, a coumarin, is a major nutrient present in noni that contributes to its antioxidative properties (e.g. Tasfiyati et al., 2022). The lifespan of *D. melanogaster* did not change when scopoletin was added to CD and it tended to be shorter when scopoletin was added to HGD (Fig. 5A). In contrast, the addition of scopoletin to HGD extended the lifespan of *D. sechellia*, similar to the effect of tetracycline (Fig. 5A). These results suggest that scopoletin contributes to the extension of *D. sechellia* lifespan, even under HGD conditions.

We further examined whether scopoletin affected gut bacterial growth and gut epithelial structure. When we cultured gut bacteria from both *D. melanogaster* and *D. sechellia* reared on HGD, scopoletin suppressed the growth of *D. melanogaster* gut bacteria cultured on BHI, LB and MRS, and it suppressed the growth of *D. sechellia* gut bacteria cultured on all studied bacterial culture media (Fig. 5B). In *D. sechellia*, the addition of scopoletin to HGD restored the disrupted gut epithelial structure, but this did not occur for *D. melanogaster* (Fig. 5C). These results indicate that scopoletin, an important component of noni, has a protective effect on survival when *D. sechellia* is fed a HGD.

## DISCUSSION

The results of multiple experiments in this study suggest that gut dysfunction in *D. sechellia* shortens its lifespan under HGD conditions. In *D. sechellia* but not *D. melanogaster* reared on HGD, the expression of genes that activate immune functions, such as the *Turandot* genes, *defensin*, *drosocin*, *metchnikowin* and some *immune-induced peptide* genes, are probably suppressed, causing failure of activation or maintenance of gut immune function. This situation would result in a change in the quantity and/or quality of gut microbiota, which can be suppressed by tetracycline or scopoletin. The change of gut microbiota seems to lead to the disorganization of gut epithelial structures and the abnormal accumulation of lipid droplets in epithelial cells.

Previous studies have uncovered robust carbohydrate-responsive regulatory systems, including mitochondrial ribosome biogenesis, intracellular calcium signaling and TGF-β/activin signaling pathways, that allow *D. melanogaster* larvae to adapt to carbohydrate-rich diets (Chng et al., 2014; Ghosh and O'Connor, 2014; Mattila et al., 2015; Melvin et al., 2018; Watanabe et al., 2019). In contrast, *D. sechellia* larvae are deficient in these systems and cannot maintain metabolic homeostasis, resulting in reduced adaptation to carbohydrate-rich diets. Therefore, it is plausible that carbohydrate-responsive regulatory systems are dysfunctional in *D. sechellia* adults. However, we found no noticeable differences in the expression of genes related to carbohydrate-responsive systems in the gut of *D. melanogaster* and *D. sechellia.* Further studies are needed to clarify the mechanistic differences in the carbohydrate-responsive systems in adults of *D. melanogaster* and *D. sechellia*, which will be crucial information for understanding the physiological differences between the adults of these two species.

We propose that high glucose levels affect gut immune function in *D. sechellia* but not *D. melanogaster*. However, the mechanistic differences between these two species remain unclear at the molecular and cellular levels. Future studies should clarify the changes in quantity and/or quality of *D. sechellia* gut microbiota between CD and HGD conditions, and how the gut microbiota change leads to the disorganization of the gut epithelium and shortened lifespan in *D. sechellia* under HGD conditions. The gut microbiota can be altered by unbalanced nutrient intake; such effects have been observed in mammals under high-carbohydrate conditions (Leeming et al., 2019; Seo et al., 2020). Therefore, it would be intriguing to examine whether an evolutionarily conserved mechanism regulates high-glucose/carbohydrate-induced immune dysfunction. As the abnormal accumulation of large lipid droplets in the gut lumen of *D. sechellia* under HGD conditions is similar to what occurs during loss of IMD function in *D. melanogaster*, we initially expected that this observation in *D. sechellia* might have the same cause. However, unlike *D. melanogaster*, there was no change in enteroendocrine Tk protein levels in *D. sechellia* reared on HGD. Unfortunately, it is technologically difficult to conduct a functional analysis of *D. sechellia* using genetic approaches to examine whether and how innate immunity pathways are involved in the high glucose-induced gut phenotypes of *D. sechellia*. However, a recent study identified some effective chemical compounds that inhibit the IMD pathway in cultured *D. melanogaster* cells (Tsukada et al., 2020). In future studies, pharmacological approaches using such chemical compounds may be more effective and could be used in functional analyses.

Interestingly, the shortened lifespan and disruption of the gut epithelial structure in flies fed HGD were restored by the addition of scopoletin, a major nutritional component of noni, to the diet. Therefore, nutrients have the potential to help *D. sechellia* survive under unbalanced nutritional conditions. This result implies that specialists can survive in unbalanced environments if their main diet or components of their main diet are present. Previous studies have largely focused on noni toxins when considering the ecological niche of *D. sechellia* and other closely related species (Jones, 2001). In contrast, our study suggests that considering the beneficial aspects of noni may also be important when investigating the differences between the specialist *D. sechellia* and generalist *Drosophila* species. It is worth noting that scopoletin extends the lifespan of *D. sechellia* but not *D. melanogaster*, whereas scopoletin tends to suppress gut bacterial growth in both *D. sechellia* and *D. melanogaster*. These results raise the possibility that scopoletin activates and improves gut immune function that specifically suppresses deleterious bacterial growth or imbalance in *D. sechellia* but not *D. melanogaster*. In future studies, it will be necessary to elucidate how scopoletin and other constituents of noni affect gut epithelial structure and gut immune function.

## Supplementary Material

10.1242/jexbio.244423_sup1 Supplementary information
