# Supplementary material for: Shortened lifespan induced by a high-glucose diet is associated with intestinal immune dysfunction in Drosophila sechellia
Source: J Exp Biol. 2022 Oct 31;225(21):jeb244423. doi: 10.1242/jeb.244423 (PMC9687539; doi:10.1242/jeb.244423)
Supplement: Supplementary information [file jexbio-225-244423-s1.pdf]

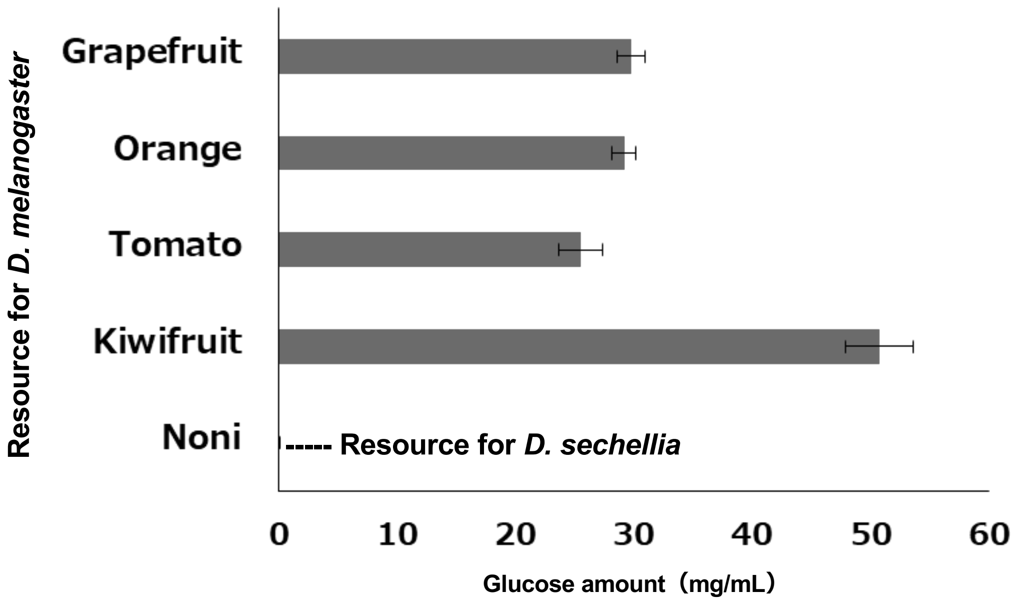

Fig. S1. Glucose concentrations present in the main diets of *D. melanogaster* and *D. sechellia*

Glucose measurement was conducted using Glucose Assay Kit (see Materials and Methods). Error bars represent standard errors of the mean (SEM). Each n=3. We confirmed that the amount of glucose in noni powder (Miracle Noni Powder 120, Nakazen Inc.) was below the detection limit of our experimental conditions. In contrast, *D. melanogaster* diets, including grapefruit, orange, tomato, and kiwifruit, contained glucose concentrations ranging from 20 to 50 mg/mL. These data are consistent with a previous report describing that noni exhibits a higher protein-to-carbohydrate ratio than the diets of *D. melanogaster* (Watanabe et al., 2019).

Journal of Experimental Biology • Supplementary information

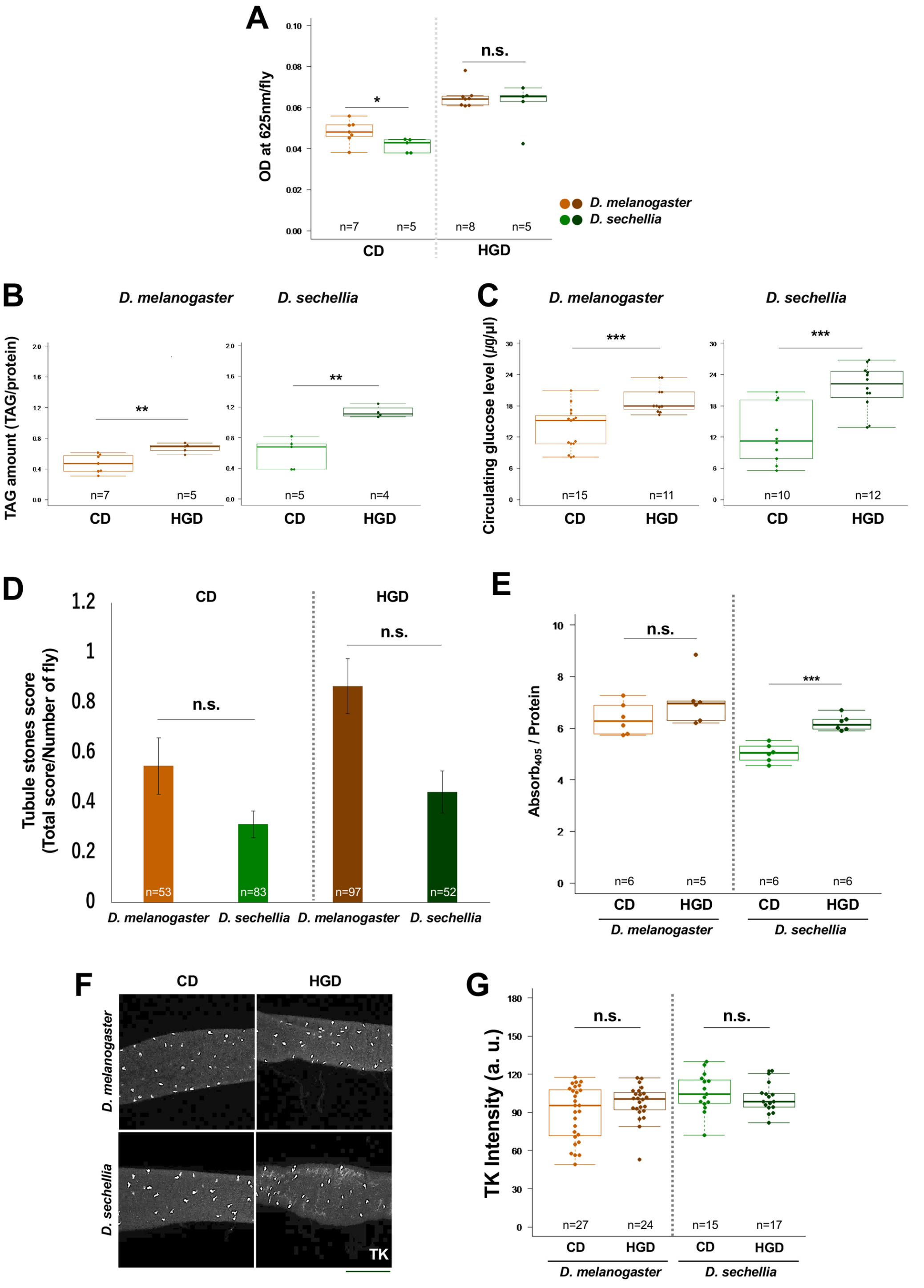
A
OD at 625nm/fly
0.10
0.08
0.06
0.04
0.02
0.00
*
n.s.
n=7
n=5
n=8
n=5
CD
HGD
D. melanogaster
D. sechellia
B
D. melanogaster
D. sechellia
TAG amount (TAG/protein)
**
**
n=7
n=5
n=5
n=4
CD
HGD
C
D. melanogaster
D. sechellia
Circulating glucose level (μg/μl)
***
***
n=15
n=11
n=10
n=12
CD
HGD
D
Tubule stones score
(Total score/Number of fly)
1.2
1
0.8
0.6
0.4
0.2
0
CD
HGD
n.s.
n.s.
n=53
n=83
n=97
n=52
D. melanogaster
D. sechellia
E
Absorb405 / Protein
10
8
6
4
2
0
n.s.
***
n=6
n=5
n=6
n=6
CD
HGD
D. melanogaster
D. sechellia
F
CD
HGD
D. melanogaster
D. sechellia
TK
G
TK Intensity (a. u.)
180
150
120
90
60
30
0
n.s.
n.s.
n=27
n=24
n=15
n=17
CD
HGD
D. melanogaster
D. sechellia

Fig. S2. Food intake, triacylglycerol (TAG) levels, circulating glucose levels, stone formation in Malpighian tubules, gut barrier function, and enteroendocrine tachykinin (Tk) levels in *D. melanogaster* and *D. sechellia* under different diet conditions.

(A) Food intake of *D. melanogaster* and *D. sechellia* under control diet (CD) and high-glucose diet (HGD) conditions. Flies were fed diets with blue dye for 24 h. Each dot corresponds to a gut lysate sample prepared by squeezing 10 guts. Box-and-whisker plots on the y-axis represent the optical densities (OD) at 625 nm normalized according to the number of gut lysate samples. Note that there was no significant difference in the absorbance between the guts of *D. melanogaster* and *D. sechellia* under the HGD condition.

(B) TAG amounts in the whole bodies of *D. melanogaster* and *D. sechellia* under CD and HGD conditions. Each dot corresponds to a whole-body lysate sample prepared by squeezing 10 virgin females. The y-axis represents the amount of TAG normalized according to the amount of protein in the whole-body lysates.

(C) Circulating glucose amounts in the body fluid of *D. melanogaster* and *D. sechellia* under CD and HGD conditions. Each dot corresponds to a hemolymph sample prepared from 30–40 adults. The y-axis represents the amount of glucose in 1 μL adult hemolymph.

(D) The degree of stone formation in the Malpighian tubules of *D. melanogaster* and *D. sechellia* under CD and HGD conditions. A five-point (from 0 to 4) scale was used to evaluate the level of stone formation in the Malpighian tubules, as previously described (van Dam et al., 2020). The y-axis represents the average score for each group divided by the number of observed Malpighian tubules. The sample numbers are presented as bars, which represent the mean ± SEM.

Journal of Experimental Biology • Supplementary information

(E) Evaluation of gut barrier function based on the activity of intestinal alkaline phosphatase (IAP), as previously described (Pereira et al., 2018). The y-axis represents the absorbance at 405 nm (Absorb405), which corresponds to the amount of dephosphorylated pNPP, normalized according to the amount of protein in each sample. (F) Enteroendocrine tachykinin (Tk) levels were unchanged between *D. melanogaster* and *D. sechellia* in CD and HGD conditions. Immunohistochemical observation of the guts of *D. melanogaster* and *D. sechellia* reared on CD and HGD. The guts were stained with anti-Tk antibody (magenta) and DAPI (blue). We focused our observations on the R2 region. Scale bar: 10 μm.

(G) Densitometric analysis of anti-Tk immunoreactivity. The y-axis represents the relative sum of anti-Tk immunostaining signals normalized by the area of the observed guts. a.u.: arbitrary unit.

*p<0.05, **p<0.001, ***p＜0.0001 (Tukey–K ramer test). n.s.: not significant.

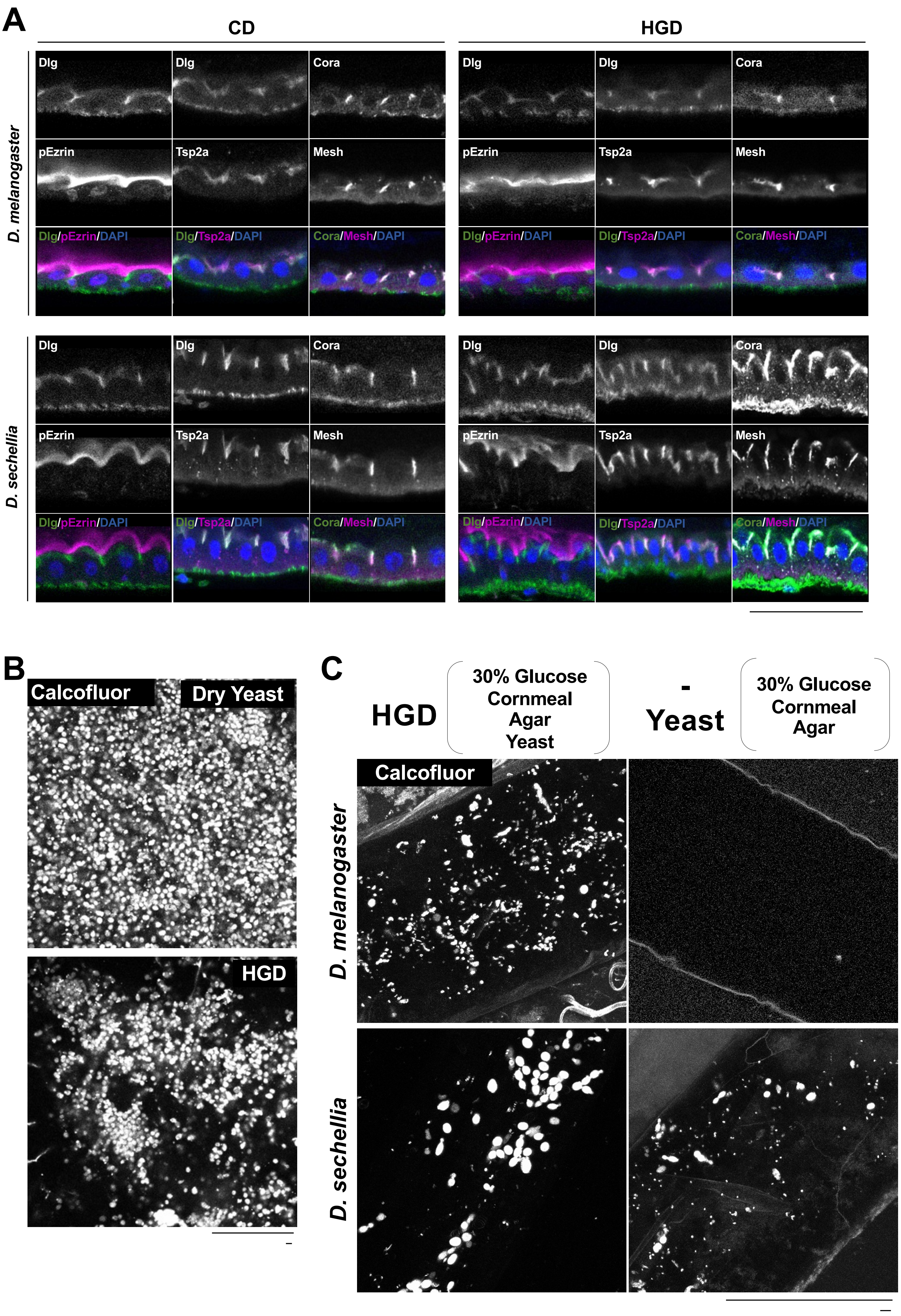

Journal of Experimental Biology • Supplementary information

Fig. S3. Gut epithelial structures of *D. melanogaster* and *D. sechellia,* and Calcofluor White staining to visualize dry yeast cell particles.

(A) Immunohistochemical observation of the gut epithelial structures of *D. melanogaster* and *D. sechellia* reared on CD and HGD. Gut epithelia were stained with DAPI (blue) and the following antibodies:

anti-Discs large (Dlg; septate junction marker; green), anti-phosphorylated Ezrin (apical marker; pEzrin; magenta), anti-Tetraspanin-2A (Tsp2a; septate junction marker; magenta), anti-Coracle (Cora; septate junction marker; green), and anti-Mesh (septate junction marker; magenta). We focused on the R2 region for our observations. The upper and lower parts of each photograph correspond to the apical and basal sides of the gut epithelium, respectively. Scale bar: 100 µm.

(B, C) Calcofluor White staining for visualization of dry yeast cell particles

(B) Calcofluor White staining of the dry yeast (dissolved in water) used for making fly food and HGD.

(C) Calcofluor White staining of bacteria, fungi, and dietary dry yeast cell particles in the gut lumen of *Drosophila* reared on HGD or a diet without dry yeast (-Yeast). Some Calcofluor White-positive large particles still remained in the -Yeast condition in *D. sechellia* gut lumens. This observation suggests that *D. sechellia* reared on HGD enriches not only dry yeast cell particles, but also some unknown large particles that differ from dry yeast cells. Long and short scale bars: 100 µm and 5 µm, respectively.

Journal of Experimental Biology • Supplementary information

Table S1. Gene expression changes between control diet and high-sugar diet in *Drosophila melanogaster*

Yellow markers indicate genes that are classified in GO:0002814.

	logFC	logCPM	PValue	FDR	FlyBase ID	Gene description
	-12.09335914	8.717014394	6.74012E-10	2.55608E-07	FBgn0032505	
	-11.04387879	7.896732588	1.76245E-09	6.2828E-07	FBgn0031176	what else
	-9.703201164	6.198922639	6.94875E-09	2.13542E-06	FBgn0085241	
	-7.879501728	4.668398999	2.81131E-08	7.1584E-06	FBgn0004429	Lysozyme P
	-7.466419263	4.10817317	4.25026E-07	8.14587E-05	FBgn0264991	
	-7.21398284	5.135087125	3.20646E-32	9.52531E-29	FBgn0020506	Amyrel
	-6.647636726	8.292906639	2.00181E-08	5.48928E-06	FBgn0028583	la costa
	-6.519839847	3.793638357	7.6021E-15	6.45237E-12	FBgn0023495	Lip3
	-6.47336865	3.326694213	8.2152E-06	0.001162125	FBgn0263763	
	-6.300414828	3.585423073	1.81192E-06	0.000291888	FBgn0263762	
	-4.899612043	4.051808103	4.36271E-16	4.86006E-13	FBgn0043005	portabella
	-4.584681335	6.747997313	1.05285E-38	1.40246E-34	FBgn0039768	
	-4.526373989	10.64722389	1.57368E-38	1.40246E-34	FBgn0051410	Niemann-Pick type C-2e
	-4.158594078	3.2184834	2.20331E-07	4.56648E-05	FBgn0040733	Bomanin S6
	-3.77369556	3.749028224	6.18618E-11	2.68933E-08	FBgn0039678	Odorant-binding protein 99a
	-3.645540085	8.74642951	8.29741E-16	8.21628E-13	FBgn0043576	Peptidoglycan recognition protein SC1a
	-3.496447887	4.350627446	2.41338E-15	2.1508E-12	FBgn0032086	
	-3.487330512	3.164517212	5.73447E-06	0.000830985	FBgn0025583	Bomanin Short 2
	-3.485182787	6.84949147	9.03303E-15	7.31839E-12	FBgn0033327	Peptidoglycan recognition protein SC1b
	-3.427530153	5.479005684	8.08958E-23	1.6021E-19	FBgn0030615	Cyp4s3
	-3.25261054	12.28722579	4.61017E-37	2.73906E-33	FBgn0028533	
	-3.243285894	3.101531451	1.96794E-05	0.00256033	FBgn0034426	Arylalkylamine N-acetyltransferase-like 5
	-3.201357236	7.124537795	1.36491E-06	0.000231696	FBgn0005391	Yolk protein 2
	-3.189019936	3.646975012	5.84765E-08	1.31935E-05	FBgn0085195	
	-3.188000452	6.783911005	1.37883E-16	1.75545E-13	FBgn0004047	Yolk protein 3
	-3.128035044	7.875020394	4.34209E-05	0.005025544	FBgn0004045	Yolk protein 1
	-3.068621094	4.762213423	1.72635E-14	1.33785E-11	FBgn0039769	
	-2.924971196	4.200442112	1.58015E-09	5.74789E-07	FBgn0022355	Transferrin 1
	-2.910632692	10.28350891	4.56209E-13	2.90409E-10	FBgn0003358	Jonah 99Ci
	-2.867866726	4.152322999	5.04564E-10	1.95508E-07	FBgn0029990	
	-2.865754912	3.490002585	1.98648E-06	0.000310588	FBgn0034331	Bomanin Bicipital 2
	-2.800472378	8.557020749	3.50461E-34	1.24932E-30	FBgn0051148	Glucocerebrosidase 1a
	-2.725603011	12.02985041	1.30483E-22	2.32573E-19	FBgn0028920	
	-2.682715599	5.106528846	8.32775E-14	6.18474E-11	FBgn0000406	Cytochrome b5-related
	-2.650647097	4.391194664	4.19228E-11	1.91598E-08	FBgn0053337	
	-2.616198955	3.947448124	4.03044E-08	9.70792E-06	FBgn0039682	Odorant-binding protein 99c
	-2.585532483	8.182658548	1.50519E-31	3.83264E-28	FBgn0038098	
	-2.563876222	8.422398532	1.97152E-34	8.7851E-31	FBgn0261675	Niemann-Pick type C-1b
	-2.562672777	3.949367021	2.40235E-08	6.4878E-06	FBgn0040606	
	-2.555767756	9.947733968	1.31281E-16	1.75545E-13	FBgn0033294	Maltase A4
	-2.513039805	5.740941472	1.50654E-07	3.19672E-05	FBgn0054040	
	-2.502635583	7.184863641	1.60933E-18	2.39039E-15	FBgn0034647	poor Imd response upon knock-in
	-2.443095987	8.500591284	2.29681E-09	8.02712E-07	FBgn0038257	Senescence marker protein-30
	-2.313813974	5.871150568	9.28343E-09	2.71259E-06	FBgn0031562	
	-2.239048957	9.35240029	3.76077E-16	4.4688E-13	FBgn0043575	Peptidoglycan recognition protein SC2
	-2.221761543	4.228422349	4.99074E-08	1.15526E-05	FBgn0001285	Jonah 44E
	-2.200381658	4.244667355	2.09022E-05	0.00269972	FBgn0032283	
	-2.178107346	3.709062155	9.18706E-06	0.001250001	FBgn0031033	
	-2.158914943	4.832155845	7.93195E-09	2.39626E-06	FBgn0032285	
	-2.069028126	5.397605837	7.27748E-11	3.08842E-08	FBgn0033792	
	-2.044433781	9.542798018	8.1064E-25	1.8061E-21	FBgn0039475	
	-1.9714217	10.92683222	2.10575E-11	1.1729E-08	FBgn0033826	
	-1.952671282	5.941217909	3.01321E-13	1.98916E-10	FBgn0039476	
	-1.916370845	8.041879906	6.24399E-09	1.95251E-06	FBgn0033999	
	-1.908805616	11.56470004	9.04556E-10	3.35892E-07	FBgn0028534	
CD>HGD	-1.828101834	9.004037329	9.3939E-21	1.52215E-17	FBgn0040256	UDP-glycosyltransferase family 35 member C1
	-1.823612291	9.240100078	3.99412E-09	1.29439E-06	FBgn0053306	
	-1.802282542	4.171970016	9.50492E-06	0.001283453	FBgn0002869	Metallothionein B
	-1.800629313	3.948858653	2.31233E-05	0.00290246	FBgn0039323	
	-1.796184938	8.861749404	4.31436E-11	1.92248E-08	FBgn0028532	
	-1.754924842	4.710319976	1.21643E-06	0.000212564	FBgn0040759	
	-1.743939564	7.087449745	6.25976E-08	1.39467E-05	FBgn0031560	
	-1.66375316	9.38912331	1.87861E-13	1.28786E-10	FBgn0262357	
	-1.655342384	6.509672201	3.1937E-05	0.003794965	FBgn0040813	
	-1.622962169	5.245137499	2.78845E-06	0.00042553	FBgn0035300	
	-1.610560149	9.615697422	1.30234E-06	0.000223201	FBgn0031561	Immune induced molecule 33
	-1.598018257	7.309309458	1.27125E-05	0.001678426	FBgn0034317	
	-1.586283739	4.150183602	7.97254E-05	0.008881413	FBgn0036262	
	-1.582329043	11.40429614	3.49117E-06	0.000522913	FBgn0036024	
	-1.565397698	4.924738894	3.82857E-06	0.000568671	FBgn0037140	SLC22A family member
	-1.547805016	9.188608402	4.64233E-16	4.86735E-13	FBgn0002571	Maltase A3

Journal of Experimental Biology • Supplementary information

	-1.542107003	8.396466211	1.04121E-11	6.18616E-09	FBgn0025454	Cyp6g1
	-1.540034569	4.594814951	1.11181E-05	0.001478874	**FBgn0028916**	
	-1.537611517	5.899551945	5.72306E-09	1.82157E-06	FBgn0033981	Cyp6a21
	-1.50319797	6.53610327	1.28495E-06	0.000222358	FBgn0032387	
	-1.49115405	5.847260863	3.21043E-08	7.9476E-06	FBgn0033093	
	-1.483947484	9.678620212	1.72931E-15	1.62228E-12	FBgn0039474	
	-1.48046697	6.642249904	3.55069E-11	1.80821E-08	FBgn0029932	
	-1.46689339	12.38412771	2.65301E-06	0.000411194	FBgn0261575	target of brain insulin
	-1.405001673	9.066738853	3.39054E-12	2.0839E-09	FBgn0039756	
	-1.384459045	6.891160559	2.38737E-10	9.89592E-08	FBgn0039349	Succinic semialdehyde dehydrogenase
	-1.383493297	5.596907745	1.47279E-06	0.000245336	FBgn0051075	
	-1.355426839	10.87156436	2.93948E-11	1.58768E-08	FBgn0033296	Maltase A7
	-1.288715435	8.273463921	2.48307E-10	1.00587E-07	FBgn0054026	
	-1.282129549	11.71456617	2.17245E-05	0.002785739	FBgn0050360	Maltase A6
	-1.277088523	7.471033423	2.79325E-06	0.00042553	FBgn0037714	
	-1.260117314	7.392784019	2.54944E-07	5.10576E-05	FBgn0086691	UK114
	-1.249626767	9.123867871	1.76943E-11	1.01736E-08	FBgn0037387	
	-1.236065759	6.477333095	4.16269E-07	8.06476E-05	FBgn0030073	
	-1.217184063	8.061443164	3.36279E-05	0.003917543	FBgn0037386	
	-1.214835028	7.511628838	6.01365E-07	0.000111653	FBgn0051300	
	-1.200413237	7.567649255	1.10166E-08	3.1671E-06	**FBgn0037906**	Peptidoglycan recognition protein LB
	-1.174262942	12.70973385	3.93848E-11	1.84736E-08	FBgn0000079	Amylase proximal
	-1.172951753	13.03090093	3.82153E-11	1.84094E-08	FBgn0000078	Amylase distal
	-1.163451857	9.570061126	1.56736E-08	4.36509E-06	FBgn0030777	
	-1.15867662	5.385983631	7.04533E-05	0.00799847	FBgn0030593	
	-1.157778219	6.920758456	2.94371E-07	5.82985E-05	FBgn0063492	Glutathione S transferase E8
	-1.153464618	5.956430867	8.81228E-06	0.001208231	FBgn0001258	Lactate dehydrogenase
	-1.149468888	10.49274449	2.91224E-05	0.003507284	FBgn0033297	Maltase A8
	-1.124375884	7.184690569	1.44928E-07	3.11228E-05	FBgn0042105	
	-1.077105033	5.599860679	7.12627E-05	0.008039158	FBgn0015240	Hormone receptor-like in 96
	-1.054220945	6.39304799	1.06781E-05	0.00143103	FBgn0024289	Sorbitol dehydrogenase 1
	-1.052628815	7.825358658	2.73386E-08	7.06206E-06	FBgn0039326	
	-1.035064362	10.34203851	6.27623E-07	0.000115327	FBgn0011834	Serine protease 6
	-1.033055687	9.134967544	6.6176E-07	0.000119144	FBgn0002569	Maltase A2
	-1.032623207	10.06481002	9.00866E-09	2.67617E-06	FBgn0030521	Cathepsin B1
	-1.028398448	9.569081907	4.85018E-08	1.13749E-05	FBgn0038037	Cyp9f2
	-1.015635771	7.60797249	2.63842E-05	0.003243257	FBgn0001089	beta galactosidase
	-1.008900855	8.958155432	3.00915E-05	0.003599667	FBgn0043783	
	-1.007067394	9.865265704	3.34718E-09	1.12566E-06	FBgn0031689	Cyp28d1
	-1.001495816	8.624554885	7.1127E-07	0.000126777	FBgn0263234	Phaedra 1
	1.022345857	9.353065282	3.34609E-05	0.003917543	FBgn0051233	
	1.114578813	8.341178973	2.32111E-07	4.72443E-05	FBgn0000044	Actin 57B
	1.191268588	6.996606701	7.13737E-06	0.001017732	FBgn0039562	Glycoprotein 93
	1.198364724	9.038395497	3.14103E-08	7.88532E-06	FBgn0001233	Heat shock protein 83
	1.198563319	6.673248338	4.08749E-06	0.000602111	FBgn0000043	Actin 42A
	1.201131249	6.536444769	5.46287E-07	0.000102495	FBgn0000565	Methionine sulfoxide reductase A
	1.230446556	6.026664282	5.19396E-06	0.000758829	FBgn0264695	Myosin heavy chain
	1.284372838	7.547263111	2.26712E-05	0.002865891	FBgn0051901	Mucin related 29B
	1.340051318	6.27058553	6.80583E-06	0.000978283	FBgn0004167	karst
	1.343189081	7.435193897	1.08675E-06	0.000191785	FBgn0001218	Heat shock 70-kDa protein cognate 3
	1.355655324	9.585690312	3.42667E-11	1.79638E-08	FBgn0259748	
	1.368628614	5.589653112	1.81775E-06	0.000291888	FBgn0000046	Actin 87E
	1.492153244	6.075135471	2.62952E-08	6.89807E-06	FBgn0031170	ATP binding cassette subfamily A
	1.570976896	4.635989912	2.69772E-05	0.003293432	FBgn0039114	Lipid storage droplet-1
CD<HGD	1.741895474	4.188138338	2.52159E-05	0.003142998	FBgn0011230	purity of essence
	1.918210232	4.837524368	8.34755E-08	1.83687E-05	FBgn0037612	
	1.989987388	8.342424063	3.73582E-11	1.84094E-08	FBgn0037782	Niemann-Pick type C-2d
	2.002150798	4.432641842	1.38633E-06	0.000233113	FBgn0030271	
	2.01130629	7.547478118	9.4933E-14	6.76834E-11	FBgn0030270	
	2.116033138	8.173880144	4.34944E-08	1.03366E-05	FBgn0027611	Lysosomal alpha-mannosidase II
	2.412991804	4.596033282	1.17246E-07	2.54852E-05	FBgn0035886	Jonah 66Ci
	2.432525026	4.442685007	3.79544E-09	1.25277E-06	FBgn0003888	beta-Tubulin at 60D
	2.568392123	8.060026059	1.40807E-08	3.98373E-06	FBgn0032068	Lysosomal alpha-mannosidase V
	2.792681643	5.367454768	1.94172E-06	0.00030901	FBgn0037996	
	2.831051083	6.498510597	5.55283E-08	1.26889E-05	FBgn0036948	
	2.914433836	8.45920852	1.76358E-07	3.69812E-05	FBgn0032069	Lysosomal alpha-mannosidase VI
	2.932611921	3.352655816	9.06345E-05	0.009972036	FBgn0052279	Drosomycin-like 2
	3.049683944	5.482847739	2.74068E-09	9.39421E-07	FBgn0053265	Mucin 68E
	3.387269681	3.565493586	2.33253E-07	4.72443E-05	FBgn0052283	Drosomycin-like 3
	4.577415534	4.257335741	1.96385E-06	0.000309767	FBgn0034407	Diptericin B

Journal of Experimental Biology • Supplementary information

Table S2. Gene expression changes between control diet and high-sugar diet in *Drosophila sechellia*

Yellow markers indicate genes that are classified in GO:0009607.

Green markers indicate genes that are classified in both GO:009607 and GO:0051607.

	logFC	logCPM	PValue	FDR	NCBI_ID	Dsec_ID	FlyBase ID of D. melanogaster putative ortholog	Gene description
	-6.545981324	5.187136396	1.88872E-08	3.71952E-06	LOC6612040	GM18691		alanine and glycine-rich protein
	-5.510454364	3.573236405	3.73522E-12	1.3096E-09	LOC116800334		FBgn0031701	Turandot M
	-5.484121966	4.982789906	2.20312E-19	2.53799E-16	LOC6612696	GM12788	FBgn0039666	protein Diedel
	-5.169978022	6.249329524	3.47257E-08	6.31579E-06	LOC6611746	GM12683	FBgn0032068	lysosomal alpha-mannosidase
	-4.909093697	5.946244473	8.15604E-16	4.38469E-13	LOC6606303	GM24207	FBgn0085345	
	-4.661931446	4.300254212	5.82891E-15	2.68596E-12	LOC6619300	GM15120	FBgn0044810	protein Turandot X1/X2
	-4.47101968	2.704302535	3.06483E-05	0.002873815	LOC6609196	GM21567	FBgn0012042	attacin-B
	-4.306602777	4.276718898	5.4466E-17	3.99285E-14	LOC6611301	GM15743		antifungal protein
	-4.281769475	4.30614688	1.48681E-17	1.40322E-14	LOC6609779	GM19909		immune-induced peptide 3
	-4.164227982	6.928451744	2.78564E-15	1.36142E-12	LOC6616986	GM10276	FBgn0051091	lipase 3
	-3.846660716	4.635720188	8.6811E-20	1.07699E-16	LOC6611287	GM14751	FBgn0261566	
	-3.775797565	6.396329442	4.22212E-13	1.62129E-10	LOC6619197	GM23143	FBgn0028396	Turandot
	-3.729497562	6.560655453	1.00035E-10	2.83045E-08	LOC6619198	GM23142	FBgn0028396	Turandot
	-3.602826032	3.338971468	1.06848E-06	0.00015386	LOC6606004	GM24370	FBgn0043578	peptidoglycan-recognition protein SB1
	-3.556709325	5.636655402	2.64996E-08	5.02807E-06	LOC6619195	GM23144	FBgn0044812	Turandot
	-3.520883076	7.082533331	7.07917E-19	7.61153E-16	LOC6617119	GM10142	FBgn0039475	phospholipase A1 VesT1.02
	-3.408333865	3.369274043	1.68451E-05	0.001705349	LOC6608609	GM20465	FBgn0033593	glycine-rich RNA-binding protein GRP1A
	-3.401822801	3.877905802	1.0091E-07	1.66068E-05	LOC6608449	GM20557	FBgn0010385	defensin
	-3.313135097	4.742699587	4.08437E-10	1.06254E-07	LOC6613407	GM18056		serine protease 1
	-3.21716907	3.501812012	6.8188E-05	0.005727789	LOC6612043	GM11543		neuropeptide-like protein 33
	-3.207864834	4.24256301	4.7357E-13	1.77622E-10	LOC6609195	GM21566	FBgn0010388	drosocin
	-3.145497937	4.674425313	7.29914E-06	0.000823499	LOC6609281	GM21609	FBgn0014865	metchnikowin
	-3.016557048	4.479105101	2.74472E-05	0.002588701	LOC6612044	GM11532		glycine-rich cell wall structural protein
	-3.013034554	8.273429444	4.55964E-12	1.53204E-09	LOC6614562	GM17281	FBgn0051789	
	-2.983901164	7.261673459	2.14687E-06	0.000286154	LOC6612042	GM11554		keratin, type II cytoskeletal 68 kDa, component IB
	-2.961152183	6.544316557	3.92615E-16	2.18348E-13	LOC6614856	GM22896	FBgn0022355	Transferrin
	-2.914225591	5.875137379	6.7287E-14	2.78258E-11	LOC6616250	GM22242	FBgn0036948	chitin binding
	-2.87188264	3.445952512	1.20759E-05	0.00128132	LOC116800660			
	-2.851786609	6.5132631	2.55387E-32	1.02972E-28	LOC6619265	GM23190		copper ion binding
	-2.842997586	10.35826775	1.87103E-35	1.09516E-31	LOC6616484	GM22392	FBgn0043783	Aldose 1-epimerase
	-2.801720151	5.222839595	4.08467E-10	1.06254E-07	LOC6614181	GM10921	FBgn0022359	sorbitol dehydrogenase
	-2.772139235	7.99348462	1.95917E-53	3.15975E-49	LOC6615296	GM15753	FBgn0034582	acidic mammalian chitinase
	-2.75910557	3.177504846	4.96074E-05	0.004335482	LOC6616100	GM19201	FBgn0023540	proteoglycan 4
	-2.74022247	4.796716804	2.77389E-15	1.36142E-12	LOC6609776	GM21868	FBgn0025583	immune-induced peptide 2
	-2.728267025	6.845804307	1.20519E-16	8.0989E-14	LOC6610437	GM14464	FBgn0035300	proton-coupled amino acid transporter-like protein CG1139
	-2.723460026	8.266534119	6.01291E-17	4.21636E-14	LOC6618457	GM17639	FBgn0004047	vitellogenin-3
	-2.675691748	3.928408298	6.55143E-07	9.87491E-05	LOC6609229	GM20167	FBgn0013772	cytochrome P450 6a8
	-2.648881616	3.653535872	4.51338E-07	6.93255E-05	LOC6615606	GM15961	FBgn0034784	putative inorganic phosphate cotransporter
	-2.551121782	8.307438385	1.97773E-23	3.9871E-20	LOC6612863	GM12878	FBgn0039768	sphingomyelin phosphodiesterase
	-2.532930354	8.053276844	2.03712E-35	1.09516E-31	LOC6617980	GM13371	FBgn0030777	serine protease SP24D
	-2.438852159	5.148704359	8.92898E-08	1.51586E-05	LOC6606685	GM24021	FBgn0010044	glutathione S-transferase 1-1
	-2.436785618	4.001290909	6.74766E-06	0.00078393	LOC6618056	GM13494	FBgn0052564	skin secretory protein xP2
	-2.430429339	6.012806804	5.5455E-14	2.35363E-11	LOC6619595	GM12005	FBgn0030615	probable cytochrome P450 4s3
	-2.42854976	7.334352486	2.4371E-08	4.67924E-06	LOC6618916	GM11771	FBgn0035089	ejaculatory bulb-specific protein 3
	-2.394349745	3.742877957	3.17408E-06	0.000403083	LOC6609777	GM21871	FBgn0040735	
	-2.392887917	7.392407587	4.52241E-15	2.14522E-12	LOC6609476	GM20043		alpha-amylase-related protein
	-2.375545494	3.288897453	0.000104507	0.008221858	LOC6609754	GM19918	FBgn0053958	
	-2.3282794	6.301383309	8.60346E-24	1.98224E-20	LOC6614734	GM16988	FBgn0032820	fructose-1,6-bisphosphatase 1
	-2.296708475	10.34446874	1.29983E-15	6.76249E-13	LOC6617912	GM13412	FBgn0286508	lambda-crystallin homolog
	-2.225753645	6.012559936	3.48527E-08	6.31579E-06	LOC6605076	GM25170	FBgn0036022	serine protease 1
	-2.203026131	4.143679421	4.15316E-06	0.000511314	LOC6615542	GM15923	FBgn0034741	
	-2.147843483	10.13084131	3.11938E-05	0.002908052	LOC6613406	GM18057		serine protease 1
	-2.127551731	11.39392428	1.90133E-29	6.13293E-26	LOC6611214	GM15309	FBgn0028534	
	-2.073614995	3.71514705	9.70812E-05	0.007867965	LOC6612845	GM12870		cecropin-A1
	-2.059451909	11.65580333	2.65217E-24	7.12904E-21	LOC116801397			putative serine protease K12H4.7
	-2.036866934	8.626974446	1.44132E-05	0.001499718	LOC6611348	GM16005	FBgn0028853	
	-2.026000408	10.39926296	5.02778E-22	8.1088E-19	LOC6609635	GM19968	FBgn0034247	facilitated trehalose transporter Tret1-2 homolog
	-1.937322217	3.923455137	2.28582E-05	0.002207528	LOC6609774	GM21867	FBgn0034330	immune-induced peptide 1
	-1.93642477	9.448890759	8.49224E-18	8.56018E-15	LOC6615446	GM15684	FBgn0054040	threonine-rich protein
	-1.931556723	7.399580432	1.98637E-22	3.55957E-19	LOC6616987	GM10277	FBgn0051089	lipase 3
	-1.905224396	9.215906863	1.56609E-17	1.40322E-14	LOC6610272	GM14230	FBgn0035189	ester hydrolase C11orf54 homolog
	-1.840965469	3.976684588	3.36174E-05	0.003080577	LOC6605497	GM24627	FBgn0036316	facilitated trehalose transporter Tret1
	-1.817549927	5.72415792	4.26083E-07	6.60757E-05	LOC6607189	GM23768	FBgn0040532	vasotab
	-1.798266853	7.04129445	1.91556E-16	1.18824E-13	LOC6608183	GM21035		maltase A3
	-1.792791018	5.259323914	6.99603E-07	0.000104474	LOC6617108	GM10152	FBgn0051075	aldehyde dehydrogenase X, mitochondrial
	-1.765193453	10.34684096	5.7784E-06	0.00068525	LOC6610956	GM13875	FBgn0035667	serine protease 1
CD>HGD	-1.742893868	8.972581522	1.48863E-08	3.07802E-06	LOC6617510	GM11404	FBgn0004045	vitellogenin-1
	-1.730947898	10.54813969	2.60279E-16	1.55473E-13	LOC116800310			activity-regulated cytoskeleton associated protein 1
	-1.71409498	3.991989311	9.84703E-05	0.007901135	LOC6618368	GM15005	FBgn0036831	
	-1.713358039	10.14110305	7.92453E-12	2.50602E-09	LOC6607072	GM26233	FBgn0037724	mucin-5AC
	-1.712290189	4.060440564	4.30072E-05	0.003832152	LOC6615304	GM15804	FBgn0034588	glycerophosphocholine phosphodiesterase GPCPD1
	-1.692350569	4.170742094	1.60606E-05	0.001639399	LOC6617117	GM10144	FBgn0039473	phospholipase A1 VesT1.02
	-1.687420977	10.63190923	8.69807E-22	1.2753E-18	LOC6617118	GM10143	FBgn0039474	phospholipase A1 VesT1.02
	-1.583425396	4.705192805	2.67822E-06	0.000348341	LOC6617538	GM11417	FBgn0030189	
	-1.550076607	12.28334033	3.1891E-17	2.44923E-14	LOC6611213	GM15045	FBgn0028920	
	-1.521287179	9.287547	1.48464E-16	9.5777E-14	LOC6611212	GM15048	FBgn0053306	
	-1.516251312	8.284627499	1.80115E-09	4.33566E-07	LOC6611292	GM15656		alcohol dehydrogenase
	-1.486699323	6.563859495	1.31626E-09	3.21647E-07	LOC6606525	GM24109	FBgn0000044	actin-87E
	-1.468913843	5.931421338	1.80145E-07	2.87662E-05	LOC6608886	GM20343	FBgn0261625	glutaminase liver isoform, mitochondrial
	-1.46854918	6.021017583	1.88799E-10	5.16092E-08	LOC6608193	GM20681	FBgn0033301	gram-negative bacteria-binding protein 3
	-1.452584228	8.028924486	1.03641E-05	0.001106971	LOC6617509	GM11339		vitellogenin-2
	-1.447135166	9.354438333	2.50645E-09	5.79665E-07	LOC6610683	GM14028	FBgn0035476	aldo-keto reductase family 1 member B1
	-1.441959361	10.12406263	2.53677E-21	3.40943E-18	LOC6609257	GM20153	FBgn0033999	lipase 3
	-1.432046078	7.009182543	3.1055E-11	8.94384E-09	LOC6615036	GM22981		glucose-6-phosphate 1-dehydrogenase
	-1.428867176	6.126804831	2.14326E-10	5.76107E-08	LOC6618276	GM23410	FBgn0032943	cytosolic 10-formyltetrahydrofolate dehydrogenase
	-1.419973408	8.080095657	7.13725E-05	0.005964229	LOC6608765	GM21337	FBgn0040759	neuropeptide-like protein 31
	-1.392761805	4.555667187	2.44116E-05	0.002343509	LOC6620109	GM13024	FBgn0087002	apolipophorins
	-1.375023699	7.307204758	7.50389E-09	1.59241E-06	LOC6615753	GM15521	FBgn0011296	protein lethal(2)essential for life
	-1.373207005	7.37304116	6.5096E-12	2.09974E-09	LOC6620989	GM26519		lysosomal acid glucosylceramidase
	-1.359704267	7.332416938	1.43049E-07	2.3304E-05	LOC6613270	GM18128	FBgn0031560	male accessory gland serine protease inhibitor
	-1.351193863	10.25854996	4.06651E-13	1.59963E-10	LOC6613088	GM18233	FBgn0031418	trans-1,2-dihydrobenzene-1,2-diol dehydrogenase
	-1.327425611	9.865132306	3.51871E-09	7.88192E-07	LOC6607230	GM23748	FBgn0037607	transketolase-like protein 2
	-1.325049818	7.221602188	5.69746E-12	1.87528E-09	LOC6606745	GM26052	FBgn0037973	L-galactose dehydrogenase
	-1.300282588	5.693151906	1.93024E-06	0.000263821	LOC6613082	GM18331	FBgn0031412	Golgi-associated plant pathogenesis-related protein 1
	-1.295872763	8.474515879	2.2762E-12	8.15791E-10	LOC6618960	GM17505	FBgn0029932	facilitated trehalose transporter Tret1
	-1.283984008	10.65635973	2.21258E-11	6.60824E-09	LOC6609755	GM21855	FBgn0034317	C-type lectin 37Da
	-1.281839962	7.586751918	1.68574E-11	5.22838E-09	LOC6615896	GM19087	FBgn0040383	mitochondrial 2-oxodicarboxylate carrier

Journal of Experimental Biology • Supplementary information

	-1.271809271	5.382076685	1.83624E-05	0.001839431	LOC6617044	GM10185	FBgn0040606	
	-1.270470725	9.021213151	3.84176E-07	6.01552E-05	LOC6617143	GM10372	FBgn0029155	NADP-dependent malic enzyme
	-1.266671911	5.172514474	4.34162E-05	0.003847342	LOC6620052	GM15067	FBgn0038858	acylpyruvase FAHD1, mitochondrial
	-1.25497086	9.73137292	8.58789E-14	3.46264E-11	LOC6617539	GM11322	FBgn0011770	putative hydroxypyruvate isomerase
	-1.247939007	6.175916177	9.08762E-08	1.52672E-05	LOC6621143	GM11136	FBgn0031533	lipase 3
	-1.237964439	7.957010459	3.99705E-08	7.16272E-06	LOC6619437	GM14750	FBgn0036927	4-aminobutyrate aminotransferase, mitochondrial
	-1.23537281	5.77070791	7.05941E-06	0.000812336	LOC6607594	GM23553	FBgn0039094	probable low-specificity L-threonine aldolase 2
	-1.23359287	10.13673466	5.34642E-10	1.3473E-07	LOC6615927	GM19006	FBgn0040349	3-oxoacyl-[acyl-carrier-protein] reductase FabG
	-1.219726267	11.06425286	3.14529E-17	2.44923E-14	LOC6611102	GM14821	FBgn0250836	acyl-CoA-binding protein
	-1.215227958	7.672593556	2.74983E-11	8.0635E-09	LOC6619276			metallothionein-3
	-1.208868523	9.119418381	4.51325E-12	1.53204E-09	LOC6616093	GM19200	FBgn0004654	6-phosphogluconate dehydrogenase, decarboxylating
	-1.1850781	7.069094799	5.70032E-07	8.67308E-05	LOC6606693	GM26021	FBgn0038020	glutathione S-transferase 1-1
	-1.174991368	7.44189287	4.68418E-09	1.03488E-06	LOC6610969	GM13871	FBgn0035679	aldose 1-epimerase
	-1.165703206	7.336330026	1.75286E-10	4.87415E-08	LOC6611382	GM16106	FBgn0028945	seminal metalloprotease 1
	-1.157235173	6.875103709	2.51591E-09	5.79665E-07	LOC6613200			fatty acid synthase
	-1.156353123	6.641897946	1.91419E-08	3.71952E-06	LOC6615866	GM19037	FBgn0023537	probable methylmalonate-semialdehyde dehydrogenase [acylating], mitochondrial
	-1.139649356	10.70620615	3.21578E-07	5.08471E-05	LOC6608187	GM20684		maltase A1
	-1.138355944	7.557367709	4.85697E-08	8.60805E-06	LOC6616943	GM10233	FBgn0039349	succinate-semialdehyde dehydrogenase [NADP(+)] GabD
	-1.132259058	5.665567478	8.39643E-06	0.000921208	LOC6617534	GM11327	FBgn0040942	
	-1.13095194	9.072095761	5.80143E-08	1.01702E-05	LOC6615809	GM16065	FBgn0023477	probable transaldolase
	-1.128481738	12.1733168	1.01398E-12	3.71672E-10	LOC6608180	GM20686		maltase A1
	-1.124880334	9.930791793	3.22992E-14	1.447E-11	LOC6608224	GM21061	**FBgn0043575**	peptidoglycan-recognition protein SC2
	-1.094251981	5.49721657	3.35719E-05	0.003080577	LOC6612009	GM18459	FBgn0027611	lysosomal alpha-mannosidase
	-1.08761492	5.792320683	7.1019E-06	0.000812336	LOC6610968	GM13872	FBgn0035678	chymotrypsin
	-1.079661733	9.813673836	2.22671E-17	1.89013E-14	LOC6611103	GM14822	FBgn0010387	acyl-CoA-binding protein homolog
	-1.07634409	5.590881549	9.04025E-05	0.007401075	LOC6614922	GM22780	FBgn0030968	L-xylulose reductase
	-1.072196455	6.799974567	1.84206E-06	0.000253921	LOC6610434	GM14463	FBgn0035298	succinyl-CoA:3-ketoacid-coenzyme A transferase, mitochondrial
	-1.053708068	5.238639574	9.80606E-05	0.007901135	LOC6611188	GM15127	**FBgn0028540**	glucose-6-phosphate 1-epimerase
	-1.051768925	6.031187377	2.22486E-05	0.002161596	LOC6613015	GM18301		muscle LIM protein 1
	-1.043093818	5.843667581	1.69181E-05	0.001705349	LOC6606689	GM24014		
	-1.042047135	7.053476243	1.89634E-08	3.71952E-06	LOC6609355	GM20101	FBgn0020236	ATP-citrate synthase
	-1.039731472	7.98183795	4.85158E-06	0.000588318	LOC6618376	GM14904	FBgn0036837	glycerophosphocholine phosphodiesterase GPCPD1
	-1.030103525	9.441536788	9.43039E-10	2.3399E-07	LOC6607368	GM23673	FBgn0038924	fumarylacetoacetate hydrolase domain-containing protein 2
	-1.011446354	9.156296217	2.02868E-11	6.17331E-09	LOC6615298	GM15801	FBgn0003748	trehalase
	-1.004146375	11.37476569	1.50208E-07	2.42255E-05	LOC6611215	GM15320	FBgn0028533	
	-1.00095979	8.121791919	2.8868E-06	0.000371454	LOC6605136	GM24822	FBgn0052054	
	1.047763713	6.463712993	2.28491E-06	0.000299876	LOC6608321	GM20618	FBgn0033391	mitochondrial folate transporter/carrier
	1.055400356	5.670593393	0.000102163	0.008084648	LOC6614674	GM17014	FBgn0032775	peroxisomal acyl-coenzyme A oxidase 3
	1.164628506	8.640828896	5.24482E-10	1.34267E-07	LOC6608766	GM20382	FBgn0085261	histidine-rich glycoprotein
	1.170308361	6.067618492	1.14321E-06	0.000163165	LOC6615182	GM23059	FBgn0031148	cystathionine beta-synthase-like protein
	1.188985182	6.552834573	6.26835E-05	0.005348988	LOC6613395	GM18500	FBgn0031645	sialin
	1.29100654	4.684379157	4.16209E-05	0.003729233	LOC6617971	GM13379	FBgn0030769	
	1.330363707	5.208893919	1.33042E-05	0.00140242	LOC6615189	GM22641	FBgn0052512	
	1.410326999	6.220525245	2.03491E-09	4.82634E-07	LOC6605607	GM24577	FBgn0036381	alanine--glyoxylate aminotransferase 2-like
	1.419174331	4.594918024	2.08992E-05	0.002063371	LOC6609097	GM21513	FBgn0033904	solute carrier family 25 member 35
CD<HGD	1.510419817	6.472448021	3.2996E-06	0.000415749	LOC6608920	GM21434	FBgn0033782	zinc finger protein GLIS2 homolog
	1.566813832	4.643158886	2.287E-06	0.000299876	LOC6616523	GM22417	FBgn0037140	organic cation transporter protein
	1.634135521	4.518303086	3.38556E-06	0.000423274	LOC6618029			proton-coupled amino acid transporter 1
	1.640627488	8.884099716	2.98795E-16	1.72106E-13	LOC6608881	GM20346	FBgn0033760	proton-coupled amino acid transporter-like protein CG1139
	1.673241195	5.673118987	8.91543E-09	1.86738E-06	LOC6616102	GM18911	FBgn0023541	probable cytochrome P450 4d14
	1.750426281	7.592201304	2.09995E-05	0.002063371	LOC6617632	GM11262	FBgn0030270	
	1.896708917	5.989759211	4.77668E-14	2.08212E-11	LOC6606719	GM26036	FBgn0037996	4-coumarate--CoA ligase 1
	1.992276739	5.976700095	7.16812E-09	1.54143E-06	LOC6618030			proton-coupled amino acid transporter 4
	2.271660062	3.849070047	2.00241E-06	0.000271386	LOC6620323	GM13255	FBgn0052656	collagen alpha-1(I) chain
	4.101275518	2.94548831	2.11096E-05	0.002063371	LOC6610577	GM14077	FBgn0035409	
